# A Robust Learning Approach for Regression Models Based on Distributionally Robust Optimization

**Published:** 2018-01-01

**Authors:** Ruidi Chen, Ioannis Ch. Paschalidis

**Affiliations:** Division of Systems Engineering, Boston University, Boston, MA 02215, USA; Department of Electrical and Computer Engineering, Division of Systems Engineering, and Department of Biomedical Engineering, Boston University, Boston, MA 02215, USA, sites.bu.edu/paschalidis

**Keywords:** Robust Learning, Distributionally Robust Optimization, Wasserstein Metric, Regularized Regression, Generalization Guarantees

## Abstract

We present a *Distributionally Robust Optimization (DRO)* approach to estimate a robustified regression plane in a linear regression setting, when the observed samples are potentially contaminated with adversarially corrupted outliers. Our approach mitigates the impact of outliers by hedging against a family of probability distributions on the observed data, some of which assign very low probabilities to the outliers. The set of distributions under consideration are close to the empirical distribution in the sense of the Wasserstein metric. We show that this DRO formulation can be relaxed to a convex optimization problem which encompasses a class of models. By selecting proper norm spaces for the Wasserstein metric, we are able to recover several commonly used regularized regression models. We provide new insights into the regularization term and give guidance on the selection of the regularization coefficient from the standpoint of a confidence region. We establish two types of performance guarantees for the solution to our formulation under mild conditions. One is related to its out-of-sample behavior (prediction bias), and the other concerns the discrepancy between the estimated and true regression planes (estimation bias). Extensive numerical results demonstrate the superiority of our approach to a host of regression models, in terms of the prediction and estimation accuracies. We also consider the application of our robust learning procedure to outlier detection, and show that our approach achieves a much higher AUC (Area Under the ROC Curve) than M-estimation (Huber, 1964, 1973).

## 1. Introduction

Consider a linear regression model with response y∈ℝ, predictor vector $x\in {\mathbb{R}}^{m-1}$, regression coefficient $\beta^{*}\in {\mathbb{R}}^{m-1}$ and error ϵ∈ℝ:

$$y=x^{\prime }\beta^{*}+\epsilon .$$

Given samples (**x**_*i*_, *y*_*i*_),*i* = 1, … , *N*, we are interested in estimating β*. The *Ordinary Least Squares (OLS)* minimizes the sum of squared residuals ${\sum_{i=1}^{N}\left(y_{i}-x_{i}^{\prime }\beta \right)}^{2}$, and works well if all the *N* samples are generated from the underlying true model. However, when faced with adversarial perturbations in the training data, the OLS estimator will deviate from the true regression plane to reduce large residuals. Alternatively, one can choose to minimize the sum of absolute residuals $\sum_{i=1}^{N}\left|y_{i}-x_{i}^{\prime }\beta \right|$, as done in *Least Absolute Deviation (LAD)*, to mitigate the influence of large residuals. Another commonly used approach for hedging against outliers is M-estimation (Huber, 1964, 1973), which minimizes a symmetric loss function *ρ*(·) of the residuals in the form $\sum_{i=1}^{N}\rho \left(y_{i}-x_{i}^{\prime }\beta \right)$, which downweights the influence of samples with large absolute residuals. Several choices for *ρ*(·) include the Huber function (Huber, 1964, 1973), the Tukey’s Biweight function (Rousseeuw and Leroy, 2005), the logistic function (Coleman et al., 1980), the Talwar function (Hinich and Talwar, 1975), and the Fair function (Fair, 1974).

Both LAD and M-estimation are not resistant to large deviations in the predictors. For contamination present in the predictor space, high breakdown value methods are required. Examples include the *Least Median of Squares (LMS)* (Rousseeuw, 1984), which minimizes the median of the absolute residuals, the *Least Trimmed Squares (LTS)* (Rousseeuw, 1985), which minimizes the sum of the *q* smallest squared residuals, and S-estimation (Rousseeuw and Yohai, 1984), which has a higher statistical efficiency than LTS with the same breakdown value. A combination of the high breakdown value method and M-estimation is the MM-estimation (Yohai, 1987). It has a higher statistical efficiency than S-estimation. We refer the reader to the book of Rousseeuw and Leroy (2005) for a detailed description of these robust regression methods.

The aforementioned robust estimation procedures focus on modifying the objective function in a heuristic way with the intent of minimizing the effect of outliers. A more rigorous line of research explores the underlying stochastic program that leads to the sample-based estimation procedures. For example, the OLS objective can be viewed as minimizing the expected squared residual under the uniform empirical distribution over the samples. It has been well recognized that optimizing under the empirical distribution yields estimators that are sensitive to perturbations in the data and suffer from overfitting. The reason is that when the data (**x**, *y*) are adversarially corrupted by outliers, the observed samples do not represent well the true underlying distribution of the data. Yet, the samples are typically the only information available. Instead of equally weighting all the samples as in the empirical distribution, we may wish to include more informative distributions that “drive out” the corrupted samples. One way to realize this is to hedge the expected loss against a family of distributions that include the true data-generating mechanism with a high confidence; an approach called *Distributionally Robust Optimization (DRO)*. DRO minimizes the worst-case expected loss over a probabilistic ambiguity set P that is constructed from the observed samples and characterized by certain known properties of the true data-generating distribution. For example, Mehrotra and Zhang (2014) study the distributionally robust least squares problem with P defined through either moment constraints, norm bounds with moment constraints, or a confidence region over a reference probability measure. Compared to the single distribution-based stochastic optimization, DRO often results in better out-of-sample performance due to its distributional robustness.

The existing literature on DRO can be split into two main branches according to the way in which P is defined. One is through a moment ambiguity set, which contains all distributions that satisfy certain moment constraints (see Popescu, 2007; Delage and Ye, 2010; Goh and Sim, 2010; Zymler et al., 2013; Wiesemann et al., 2014). In many cases, it leads to a tractable DRO problem but has been criticized for yielding overly conservative solutions (Wang et al., 2016). The other is to define P as a ball of distributions using some probabilistic distance functions such as the *ϕ*-divergences (Bayraksan and Love, 2015), which include the Kullback-Leibler (KL) divergence (Hu and Hong, 2013; Jiang and Guan, 2015) as a special case, the Prokhorov metric (Erdoğan and Iyengar, 2006), and the Wasserstein distance (Esfahani and Kuhn, 2015; Gao and Kleywegt, 2016; Zhao and Guan, 2015; Luo and Mehrotra, 2017; Blanchet and Murthy, 2016). Deviating from the stochastic setting, there are also some works focusing on deterministic robustness. El Ghaoui and Lebret (1997) consider the least squares problem with unknown but bounded, non-random disturbance and solve it in polynomial time. Xu et al. (2010) study the robust linear regression problem with norm-bounded feature perturbation and show that it is equivalent to the *ℓ*_1_-regularized regression. See Yang and Xu (2013); Bertsimas and Copenhaver (2017) which also use a deterministic robustness approach.

In this paper we consider a DRO problem with P containing distributions that are close to the discrete empirical distribution in the sense of Wasserstein distance. The reason for choosing the Wasserstein metric is two-fold. On one hand, the Wasserstein ambiguity set is rich enough to contain both continuous and discrete relevant distributions, while other metrics such as the KL divergence, exclude all continuous distributions if the nominal distribution is discrete (Esfahani and Kuhn, 2015; Gao and Kleywegt, 2016). Furthermore, considering distributions within a KL distance from the empirical, does not allow for probability mass outside the support of the empirical distribution. On the other hand, measure concentration results guarantee that the Wasserstein set contains the true data-generating distribution with high confidence for a sufficiently large sample size (Fournier and Guillin, 2015). Moreover, the Wasserstein metric takes into account the closeness between support points while other metrics such as the *ϕ*-divergence only consider the probabilities of these points. The image retrieval example in Gao and Kleywegt (2016) suggests that the probabilistic ambiguity set constructed based on the KL divergence prefers the pathological distribution to the true distribution, whereas the Wasserstein distance does not exhibit such a problem. The reason lies in that *ϕ*-divergence does not incorporate a notion of closeness between two points, which in the context of image retrieval represents the perceptual similarity in color.

Our DRO problem minimizes the worst-case absolute residual over a Wasserstein ball of distributions, and could be relaxed to the following form:

(1) $$\underset{\beta }{\text{inf}}\frac{1}{N}\sum_{i=1}^{N}\left|y_{i}-x_{i}^{\prime }\beta \right|+\epsilon ∥(-\beta ,1){∥}_{*},$$

where *ϵ* is the radius of the Wasserstein ball, and ∥ · ∥_*_ is the dual norm of the norm space where the Wasserstein metric is defined on. Formulation (1) incorporates a wide class of models whose specific form depends on the notion of transportation cost embedded in the Wasserstein metric (see Section 2). Although the Wasserstein DRO formulation simply reduces to regularized regression models, we want to emphasize a few new insights brought by this methodology. First, the regularization term controls the conservativeness of the Wasserstein set, or the amount of ambiguity in the data, which differentiates itself from the heuristically added regularizers in traditional regression models that serve the purpose of preventing overfitting, error/variance reduction, or sparsity recovery. Second, the regularization term is determined by the dual norm of the regression coefficient, which controls the *growth rate* of the *ℓ*_1_-loss function, and the radius of the Wasserstein set. This connection provides guidance on the selection of the regularization coefficient and may lead to significant computational savings compared to cross-validation. DRO essentially enables new and more accurate interpretations of the regularizer, and establishes its dependence on the *growth rate* of the loss, the underlying metric space and the reliability of the observed samples.

The connection between robustness and regularization has been established in several works. The earliest one may be credited to El Ghaoui and Lebret (1997), who show that minimizing the worst-case squared residual within a Frobenius norm-based perturbation set is equivalent to Tikhonov regularization. In more recent works, using properly selected uncertainty sets, Xu et al. (2010) have shown the equivalence between robust linear regression with feature perturbations and the *Least Absolute Shrinkage and Selection Operator (LASSO)*. Yang and Xu (2013) extend this to more general LASSO-like procedures, including versions of the grouped LASSO. Bertsimas and Copenhaver (2017) give a comprehensive characterization of the conditions under which robustification and regularization are equivalent for regression models with deterministic norm-bounded perturbations on the features. For classification problems, Xu et al. (2009) show the equivalence between the regularized Support Vector Machines (SVMs) and a robust optimization formulation, by allowing potentially correlated disturbances in the covariates.Shafieezadeh-Abadeh et al. (2015) consider a robust version of logistic regression under the assumption that the probability distributions under consideration lie in a Wasserstein ball, and they show that the regularized logistic regression is a special case of this robust formulation. Recently,Shafieezadeh-Abadeh et al. (2017); Gao et al. (2017) have provided a unified framework for connecting the Wasserstein DRO with regularized learning procedures, for various regression and classification models.

Our work is motivated by the problem of identifying patients who receive an abnormally high radiation exposure in CT exams, given the patient characteristics and exam-related variables (Chen et al., 2018). This could be casted as an outlier detection problem; specifically, estimating a robustified regression plane that is immunized against outliers and learns the underlying true relationship between radiation dose and the relevant predictors. We focus on robust learning of the parameter in regression models under distributional perturbations residing within a Wasserstein ball. While the applicability of the Wasserstein DRO methodology is not restricted to regression analysis (Sinha et al., 2017; Gao et al., 2017;Shafieezadeh-Abadeh et al., 2017), or a particular form of the loss function (as long as it satisfies certain smoothness conditions (Gao et al., 2017)), we focus on the absolute residual loss in linear regression in light of our motivating application and for the purpose of enhancing robustness. Our contributions can be summarized as follows:

1. We develop a DRO approach to robustify linear regression using an *ℓ*_1_ loss function and an ambiguity set around the empirical distribution of the training samples defined based on the Wasserstein metric. The formulation is general enough to include any norm-induced Wasserstein metric and incorporate additional regularization constraints on the regression coefficients (e.g., *ℓ*_1_-norm constraints). It provides an intuitive connection between the amount of ambiguity allowed and a regularization penalty term in the robust formulation, which provides a natural way to adjust the latter.
2. We establish novel performance guarantees on both the out-of-sample loss (predictionbias) and the discrepancy between the estimated and the true regression coefficients (estimation bias). Our guarantees elucidate the role of the regularizer, which is related to the dual norm of the regression coefficients, in bounding the biases and are in concert with the theoretical foundation that leads to the regularized problem. The generalization error bound, in particular, builds a connection between the loss function and the form of the regularizer via Rademacher complexity, providing a rigorous explanation for the commonly observed good out-of-sample performance of regularized regression. On the other hand, the estimation error bound corroborates the validity of the *ℓ*_1_-loss function, which tends to incur a lower estimation bias than other candidates such as the *ℓ*_2_ and *ℓ*_∞_ losses. Our results are novel in the robust regression setting and different from earlier work in the DRO literature, enabling new perspectives and interpretations of the norm-based regularization, and providing justifications for the *ℓ*_1_-loss-based learning algorithms.
3. We empirically explore three important aspects of the Wasserstein DRO formulation, including the advantages of the *ℓ*_1_-loss function, the selection of a proper norm for the Wasserstein metric, and the implication of penalizing the *extended regression coefficient* (−***β***, 1), by comparing with a series of regression models on a number of synthetic datasets. We show the superiority of the Wasserstein DRO approach, presenting a thorough analysis under four different experimental setups. We also consider the application of our methodology to outlier detection and compare with M-estimation in terms of the ability of identifying outliers (*ROC (Receiver Operating Characteristic)* curves). The Wasserstein DRO formulation achieves significantly higher *AUC (Area Under Curve)* values.

The rest of the paper is organized as follows. In Section 2, we introduce the Wasserstein metric and derive the general Wasserstein DRO formulation in a linear regression framework. Section 3 establishes performance guarantees for both the general formulation and the special case where the Wasserstein metric is defined on the *ℓ*_1_-norm space. Numerical experimental results are presented in Section 4. We conclude the paper in Section 5.

### Notational conventions:

We use boldfaced lowercase letters to denote vectors, ordinary lowercase letters to denote scalars, boldfaced uppercase letters to denote matrices, and calligraphic capital letters to denote sets. E denotes expectation and ℙ probability of an event. All vectors are column vectors. For space saving reasons, we write **x** = (*x*_1_, … , *x*_dim(**x**)_) to denote the column vector **x**, where dim(**x**) is the dimension of **x**. We use prime to denote the transpose of a vector, ∥·∥ for the general norm operator, ∥·∥_2_ for the *ℓ*_2_ norm, ∥ · ∥_1_ for the *ℓ*_1_ norm, and ∥ · ∥_∞_ for the infinity norm. P(Z) denotes the set of probability measures supported on Z. **e**_*i*_ denotes the *i*-th unit vector, **e** the vector of ones, **0** a vector of zeros, and **I** the identity matrix. Given a norm ∥ · ∥ on ${\mathbb{R}}^{m}$, the dual norm ∥ · ∥_*_ is defined as: ∥***θ***∥_*_ ≜ sup_∥**z**∥≤1_ ***θ***′**z**. For a function *h*(**z**), its convex conjugate h*(·) is defined as: h*(***θ***) ≜ sup_**z**∈dom *h*_ {***θ***′**z** − *h*(**z**)}, where dom *h* denotes the domain of the function *h*.

## 2. Problem Statement and Justification of Our Formulation

**Figure 1:**
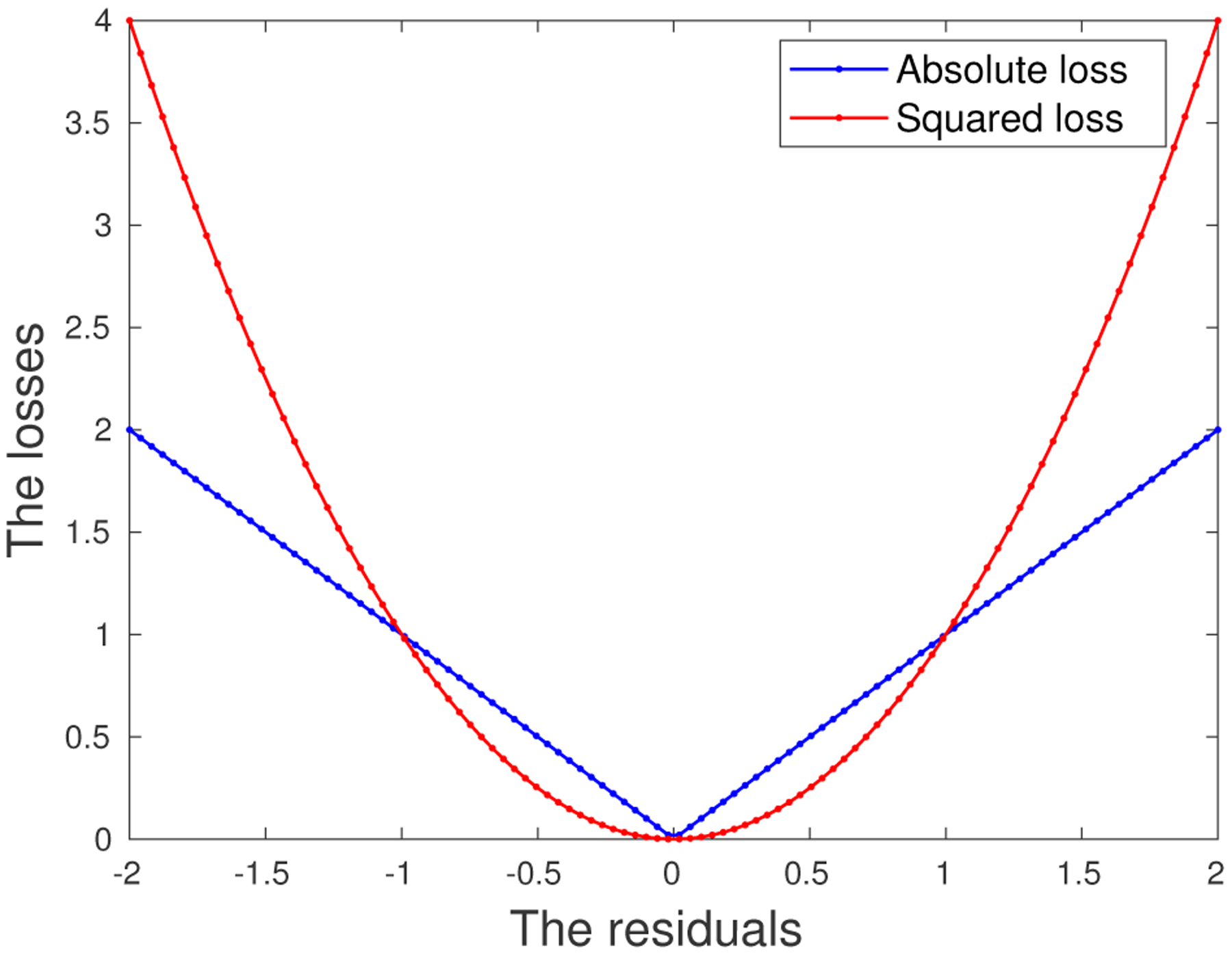
Comparison between *ℓ*_1_ and *ℓ*_2_ loss functions.

Consider a linear regression problem where we are given a predictor/feature vector $x\in {\mathbb{R}}^{m-1}$, and a response variable y∈ℝ. Our goal is to obtain an accurate estimate of the regression plane that is robust with respect to the adversarial perturbations in the data. We consider an *ℓ*_1_-loss function *h*_***β***_(**x**, *y*) ≜ |*y* − **x**′***β***|, motivated by the observation that the absolute loss function is more robust to large residuals than the squared loss (see Fig. 1). Moreover, the estimation error analysis presented in Section 3.2 suggests that the *ℓ*_1_-loss function leads to a smaller estimation bias than others. Our Wasserstein DRO problem using the *ℓ*_1_-loss function is formulated as:

(2) $$\underset{\beta \in B}{\text{inf}}\underset{\mathbb{Q}\in \Omega }{\text{sup}}E^{\mathbb{Q}}\left[\left|y-x^{\prime }\beta \right|\right],$$

where ***β*** is the regression coefficient vector that belongs to some set B. B could be ${\mathbb{R}}^{m-1}$, or $B=\left\{\beta :\|\beta {\|}_{1}\leq l\right\}$ if we wish to induce sparsity, with *l* being some pre-specified number. ℚ is the probability distribution of (**x**, *y*), belonging to some set Ω which is defined as:

$$\Omega ≜\left\{\mathbb{Q}\in P(Z):W_{p}\left(\mathbb{Q},\;{\hat{\mathbb{P}}}_{N}\right)\leq \epsilon \right\},$$

where Z is the set of possible values for (**x**, *y*); P(Z) is the space of all probability distributions supported on is Z; *ϵ* a pre-specified radius of the Wasserstein ball; and $W_{p}\left(\mathbb{Q},{\hat{\mathbb{P}}}_{N}\right)$ is the order-*p* Wasserstein distance between ℚ and ${\hat{\mathbb{P}}}_{N}$ (see definition in (3)), with ${\hat{\mathbb{P}}}_{N}$ the uniform empirical distribution over samples. The formulation in (2) is robust since it minimizes over the regression coefficients the worst case expected loss, that is, the expected loss maximized over all probability distributions in the ambiguity set Ω.

Before deriving a tractable reformulation for (2), let us first define the Wasserstein metric. Let (Z, *s*) be a metric space where Z is a set and *s* is a metric on Z. The Wasserstein metric of order *p* ≥ 1 defines the distance between two probability distributions ${\mathbb{Q}}_{1}$ and ${\mathbb{Q}}_{2}$ in the following way:

(3) $$W_{p}\left({\mathbb{Q}}_{1},{\mathbb{Q}}_{2}\right)≜{\left(\underset{\text{Π}\in P(Z\times Z)}{\text{min}}\left\{\int_{Z\times Z}{\left(s\left(\left(x_{1},y_{1}\right),\left(x_{2},y_{2}\right)\right)\right)}^{p}\text{Π}\left(d\left(x_{1},y_{1}\right),d\left(x_{2},y_{2}\right)\right)\right\}\right)}^{1/p},$$

where Π is the joint distribution of (**x**_1_, *y*_1_) and (**x**_2_, *y*_2_) with marginals ${\mathbb{Q}}_{1}$ and ${\mathbb{Q}}_{2}$, respectively. The Wasserstein distance between ${\mathbb{Q}}_{1}$ and ${\mathbb{Q}}_{2}$ represents the cost of an optimal mass transportation plan, where the cost is measured through the metric *s*. The order *p* should be selected in such a way as to ensure that the worst-case expected loss is meaningfully defined, i.e.,

(4) $$E^{\mathbb{Q}}\left[h_{\beta }(x,y)\right]<\infty ,\;\forall \mathbb{Q}\in \Omega \text{.}$$

Notice that the ambiguity set Ω is centered at the empirical distribution ${\hat{\mathbb{P}}}_{N}$ and has radius *ϵ*. It may be desirable to translate (4) into:

(5) $$\left|E^{\mathbb{Q}}\left[h_{\beta }(x,y)\right]-E^{{\hat{\mathbb{P}}}_{N}}\left[h_{\beta }(x,y)\right]\right|<\infty ,\forall \mathbb{Q}\in \Omega \text{.}$$

We want to relate (5) with the Wasserstein distance $W_{p}\left(\mathbb{Q},{\hat{\mathbb{P}}}_{N}\right)$, which is no larger than for all ℚ∈Ω. The LHS of (5) could be written as:

(6) $$\left|E^{\mathbb{Q}}\left[h_{\beta }(x,y)\right]-E^{{\hat{\mathbb{P}}}_{N}}\left[h_{\beta }(x,y)\right]\right|\;=\left|\int_{Z}h_{\beta }\left(x_{1},y_{1}\right)\mathbb{Q}\left(d\left(x_{1},y_{1}\right)\right)-\int_{Z}h_{\beta }\left(x_{2},y_{2}\right){\hat{\mathbb{P}}}_{N}\left(d\left(x_{2},y_{2}\right)\right)\right|\;=\left|\int_{Z}h_{\beta }\left(x_{1},y_{1}\right)\int_{Z}{\text{Π}}_{0}\left(d\left(x_{1},y_{1}\right),d\left(x_{2},y_{2}\right)\right)-\int_{Z}h_{\beta }\left(x_{2},y_{2}\right)\int_{Z}{\text{Π}}_{0}\left(d\left(x_{1},y_{1}\right),d\left(x_{2},y_{2}\right)\right)\right|\;\leq \int_{Z\times Z}\left|h_{\beta }\left(x_{1},y_{1}\right)-\right|h_{\beta }\left(x_{2},y_{2}\right)|{\text{Π}}_{0}\left(d\left(x_{1},y_{1}\right),d\left(x_{2},y_{2}\right)\right),$$

where Π_0_ is the joint distribution of (**x**_1_, *y*_1_) and (**x**_2_, *y*_2_) with marginals ℚ and ${\hat{\mathbb{P}}}_{N}$, respectively. Comparing (6) with (3), we see that for (5) to hold, the following quantity which characterizes the *growth rate* of the loss function needs to be bounded:

(7) $${\text{GR}}_{h_{\beta }}\left(\left(x_{1},y_{1}\right),\left(x_{2},y_{2}\right)\right)≜\frac{\left|h_{\beta }\left(x_{1},y_{1}\right)-h_{\beta }\left(x_{2},y_{2}\right)\right|}{{\left(s\left(\left(x_{1},y_{1}\right),\left(x_{2},y_{2}\right)\right)\right)}^{p}},\forall \left(x_{1},y_{1}\right),\left(x_{2},y_{2}\right)\in Z.$$

A formal definition of the growth rate is due to Gao and Kleywegt (2016), which takes the limit of (7) as *s*((**x**_1_, *y*_1_),(**x**_2_, *y*_2_)) → ∞, to eliminate its dependence on (**x**, *y*). One important aspect they have pointed out is that when the growth rate of the loss function is infinite, strong duality for the worst-case problem ${\text{sup}}_{\mathbb{Q}\in \Omega }E^{\mathbb{Q}}\left[h_{\beta }(x,y)\right]$ fails to hold, in which case the DRO problem (2) becomes intractable. Assuming that the metric *s* is induced by some norm ∥ · ∥, the bounded *growth rate* requirement is expressed as follows:

(8) $$\underset{\left\|\left(x_{1},y_{1}\right)-\left(x_{2},y_{2}\right)\right\|\to \infty }{\text{lim}\;\text{sup}}\frac{\left|h_{\beta }\left(x_{1},y_{1}\right)-h_{\beta }\left(x_{2},y_{2}\right)\right|}{{\left\|\left(x_{1},y_{1}\right)-\left(x_{2},y_{2}\right)\right\|}^{p}}\leq \underset{\left\|\left(x_{1},y_{1}\right)-\left(x_{2},y_{2}\right)\right\|\to \infty }{\text{lim}\;\text{sup}}\frac{\left|y_{1}-x_{1}^{\prime }\beta -\left(y_{2}-x_{2}^{\prime }\beta \right)\right|}{{\left\|\left(x_{1},y_{1}\right)-\left(x_{2},y_{2}\right)\right\|}^{p}}\leq \underset{\left\|\left(x_{1},y_{1}\right)-\left(x_{2},y_{2}\right)\right\|\to \infty }{\text{lim}\;\text{sup}}\frac{\left\|\left(x_{1},y_{1}\right)-\left(x_{2},y_{2}\right)\right\|{\left\|\left(-\beta ,1\right)\right\|}_{*}}{{\left\|\left(x_{1},y_{1}\right)-\left(x_{2},y_{2}\right)\right\|}^{p}}<\infty ,$$

where ∥·∥_*_ is the dual norm of ∥·∥, and the second inequality is due to the Cauchy-Schwarz inequality. Notice that by taking *p* = 1, (8) is equivalently translated into the condition that ∥(−***β***, 1)∥_*_ < ∞, which we will see in Section 3 is an essential requirement to guarantee a good generalization performance for the Wasserstein DRO estimator. The growth rate essentially reveals the underlying metric space used by the Wasserstein distance. Taking *p* > 1 leads to zero growth rate in the limit of (8), which is not desirable since it removes the Wasserstein ball structure from our formulation and renders it an optimization problem over a singleton distribution. This will be made more clear in the following analysis. We thus choose the order-1 Wasserstein metric with *s* being induced by some norm ∥ · ∥ to define our DRO problem.

**Figure 2:**
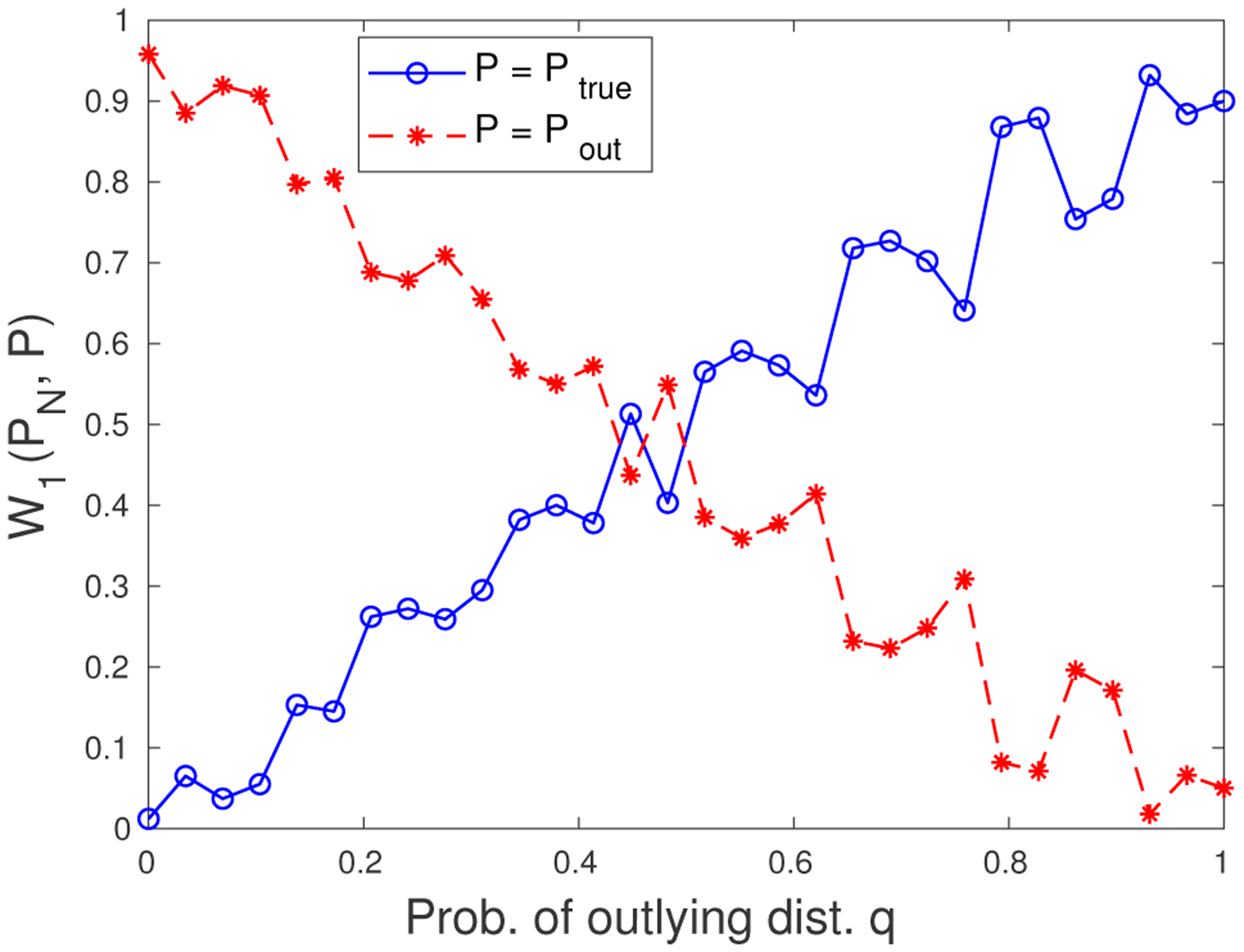
The order-1 Wasserstein distances from the empirical distribution.

Next, we will discuss how to convert (2) into a tractable formulation. Suppose we have *N* independently and identically distributed realizations of (**x**, *y*), denoted by (**x**_*i*_, *y*_*i*_), *i* = 1, … , *N*. We make the assumption that (**x**, *y*) comes from a mixture of two distributions, with probability *q* from the outlying distribution ${\mathbb{P}}_{\text{out}}$ and with probability 1 − *q* from the true distribution ℙ. Recall that ${\hat{\mathbb{P}}}_{N}$ is the discrete uniform distribution over the *N* samples. Our goal is to generate estimators that are consistent with the true distribution ℙ. We claim that when *q* is small, if the Wasserstein ball radius *ϵ* is chosen judiciously, the true distribution ℙ will be included in the set Ω while the outlying distribution ${\mathbb{P}}_{out}$ will be excluded. To see this, consider a simple example where ℙ is a discrete distribution that assigns equal probability to 10 data points equally spaced between 0.1 and 1, and ${\mathbb{P}}_{out}$ assigns probability 0.5 to two data points 1 and 2. We generate 100 samples and plot the Wasserstein distances from ${\hat{\mathbb{P}}}_{N}$ for both ℙ and ${\mathbb{P}}_{out}$. From Fig. 2 we observe that for *q* below 0.5, the true distribution ℙ is closer to ${\hat{\mathbb{P}}}_{N}$ whereas the outlying distribution ${\mathbb{P}}_{out}$ is further away. If the radius *ϵ* is chosen between the red (*−) and blue (⟜) lines, the Wasserstein ball that we are hedging against will exclude the outlying distribution and the resulting estimator will be robust to the adversarial perturbations. Moreover, as *q* becomes smaller, the gap between the red and blue lines becomes larger. One implication from this observation is that as the data becomes purer, the radius of the Wasserstein ball tends to be smaller, and the confidence in the observed samples is higher. For large *q* values, the DRO formulation seems to fail. However, as outliers are defined to be the data points that do not conform to the majority of data, we can safely claim that ${\mathbb{P}}_{out}$ is the distribution of the minority and *q* is always below 0.5.

We now consider the inner supremum in (2). Esfahani and Kuhn (2015, Theorem 6.3) show that when the set Z is closed and convex, and the loss function *h*_***β***_(**x**, *y*) is convex in (**x**, *y*),

(9) $$\underset{\mathbb{Q}\in \Omega }{\text{sup}}E^{\mathbb{Q}}\left[h_{\beta }(x,y)\right]\leq \kappa \epsilon +\frac{1}{N}\sum_{i=1}^{N}h_{\beta }\left(x_{i},y_{i}\right),\forall \epsilon \geq 0,$$

Where $\kappa (\beta )=\text{sup}\left\{\|\theta {\|}_{*}:h_{\beta }^{*}(\theta )<\infty \right\}$, with $h_{\beta }^{*}(\cdot )$ the convex conjugate function of *h*_***β***_(**x**, *y*). Through (9), we can relax problem (2) by minimizing the right hand side of (9) instead of the worst-case expected loss. Moreover, as shown in Esfahani and Kuhn (2015), (9) becomes an equality when $Z={\mathbb{R}}^{m}$. In Theorem 2.1, we compute the value of *κ*(***β***) for the specific *ℓ*_1_ loss function we use. The proof of this Theorem and all results hereafter are included in Appendix A.

### Theorem 2.1

Define $\kappa (\beta )=\text{sup}\left\{\|\theta {\|}_{*}:h_{\beta }^{*}(\theta )<\infty \right\}$ where ∥·∥_*_ is the dual norm of ∥·∥, and $h_{\beta }^{*}(\cdot )$ is the conjugate function of *h*_*β*_(·). When the loss function is *h*_***β***_(**x**, *y*) = |*y* − **x**′***β***|, we have *κ*(***β***) = ∥(−***β***, 1) ∥_*_.

Due to Theorem 2.1, (2) could be formulated as the following optimization problem:

(10) $$\underset{\beta \in B}{\text{inf}}\epsilon {\left\|\left(-\beta ,1\right)\right\|}_{*}+\frac{1}{N}\sum_{i=1}^{N}\left|y_{i}-x_{i}^{\prime }\beta \right|.$$

Note that the regularization term of (10) is the product of the *growth rate* of the loss and the Wasserstein ball radius. The growth rate is closely related to the way the Wasserstein metric defines the transportation costs on the data (**x**, *y*). As mentioned earlier, a zero growth rate diminishes the effect of the Wasserstein distributional uncertainty set, and the resulting formulation would simply be an empirical loss minimization problem. The parameter *ϵ* controls the conservativeness of the formulation, whose selection depends on the sample size, the dimensionality of the data, and the confidence that the Wasserstein ball contains the true distribution (see eq. (8) in Esfahani and Kuhn, 2015). Roughly speaking, when the sample size is large enough, and for a fixed confidence level, *ϵ* is inversely proportional to *N*^1/*m*^.

Formulation (10) incorporates a class of models whose specific form depends on the norm space we choose, which could be application-dependent and practically useful. For example, when the Wasserstein metric *s* is induced by ∥ · ∥_2_ and the set B is the intersection of a polyhedron with convex quadratic inequalities, (10) is a convex quadratic problem which can be solved to optimality very efficiently. Specifically, it could be converted to:

(11) $$\underset{a,b_{1},\ldots ,b_{N},\beta }{\text{min}}a\epsilon +\frac{1}{N}\sum_{i=1}^{N}b_{i}\;\text{s.t.}\;{\left\|\beta \right\|}_{2}^{2}+1\leq a^{2},\;y_{i}-x_{i}^{\prime }\beta \leq b_{i},i=1,\ldots ,N,\;-\left(y_{i}-x_{i}^{\prime }\beta \right)\leq b_{i},i=1,\ldots ,N,\;a,b_{i}\geq 0,i=1,\ldots ,N,\;\beta \in B\text{.}$$

When the Wasserstein metric is defined using ∥·∥_1_ and the set B is a polyhedron, (10) is a linear programming problem:

(12) $$\underset{a,b_{1},\ldots ,b_{N},\beta }{\text{min}}a\epsilon +\frac{1}{N}\sum_{i=1}^{N}b_{i}\;\text{s.t.}\;a\geq \beta^{\prime }e_{i},\;i=1,\ldots ,m-1\text{,}\;a\geq -\beta^{\prime }e_{i},i=1,\ldots ,m-1,\;y_{i}-x_{i}^{\prime }\beta \leq b_{i},i=1,\ldots ,N,\;-\left(y_{i}-x_{i}^{\prime }\beta \right)\leq b_{i},i=1,\ldots ,N,\;a\geq 1\text{,}\;b_{i}\geq 0,i=1,\ldots ,N,\;\beta \in B\text{.}$$

More generally, when the coordinates of (**x**, *y*) differ from each other substantially, a properly chosen, positive definite weight matrix $M\in {\mathbb{R}}^{m\times m}$ could scale correspondingly different coordinates of (**x**, *y*) by using the **M**-weighted norm:

$${\left\|\left(x,y\right)\right\|}_{\text{M}}=\sqrt{\left(x,y{)}^{\prime }M(x,y\right)}.$$

It can be shown that (10) in this case becomes:

(13) $$\underset{\beta \in B}{\text{inf}}\epsilon \sqrt{\left(-\beta ,1{)}^{\prime }M^{-1}(-\beta ,1\right)}+\frac{1}{N}\sum_{i=1}^{N}\left|y_{i}-x_{i}^{\prime }\beta \right|.$$

We note that this Wasserstein DRO framework could be applied to a broad class of loss functions and the tractable reformulations have been derived in Shafieezadeh-Abadeh et al. (2017); Gao et al. (2017) for regression and classification models. We adopt the absolute residual loss in this paper to enhance the robustness of the formulation, which is the focus of our work and serves the purpose of estimating robust parameters that are immunized against perturbations/outliers. Notice that (10) coincides with the regularized LAD models (Pollard, 1991; Wang et al., 2006), except that we are regularizing a variant of the regression coefficient. We would like to highlight several novel viewpoints that are brought by the Wasserstein DRO framework and justify the value and novelty of (10). First, (10) is obtained as an outcome of a fundamental DRO formulation, which enables new interpretations of the regularizer from the standpoint of distributional robustness, and provides rigorous theoretical foundation on why the *ℓ*_2_-regularizer prevents overfitting to the training data. The regularizer could be seen as a control over the amount of ambiguity in the data and reveals the reliability of the contaminated samples. Second, the geometry of the Wasserstein ball is embedded in the regularization term, which penalizes the regression coefficient on the dual Wasserstein space, with the magnitude of penalty being the radius of the ball. This offers an intuitive interpretation and provides guidance on how to set the regularization coefficient. Moreover, different from the traditional regularized LAD models that directly penalize the regression coefficient ***β***, we regularize the vector (−***β***, 1), where the 1 takes into account the transportation cost along the *y* direction. Penalizing only on ***β*** corresponds to an infinite transportation cost along *y*. Our model is more general in this sense, and establishes the connection between the metric space on data and the form of the regularizer.

## 3. Performance Guarantees

Having obtained a tractable reformulation for the Wasserstein DRO problem, we next establish guarantees on the predictive power and estimation quality for the solution to (10). Two types of results will be presented in this section, one of which bounds the prediction bias of the estimator on new, future data (given in Section 3.1). The other one that bounds the discrepancy between the estimated and true regression planes (estimation bias), is given in Section 3.2.

### 3.1 Out-of-Sample Performance

In this subsection we investigate generalization characteristics of the solution to (10), which involves measuring the error generated by our estimator on a new random sample (**x**, *y*). We would like to obtain estimates that not only explain the observed samples well, but, more importantly, possess strong generalization abilities. The derivation is mainly based on *Rademacher complexity* (see Bartlett and Mendelson, 2002), which is a measurement of the complexity of a class of functions. We would like to emphasize the applicability of such a proof technique to general loss functions, as long as their empirical Rademacher complexity could be bounded. The bound we derive for the prediction bias depends on both the sample average loss (the training error) and the dual norm of the regression coefficient (the regularizer), which corroborates the validity and necessity of our regularized formulation. Moreover, the generalization result also builds a connection between the loss function and the form of the regularizer via Rademacher complexity, which enables new insights into the regularization term and explains the commonly observed good out-of-sample performance of regularized regression in a rigorous way. We first make several mild assumptions that are needed for the generalization result.

#### Assumption A

The norm of the uncertainty parameter (**x**, *y*) is bounded above almost surely, i.e., ∥(**x**, *y*)∥ ≤ *R*.

#### Assumption B

The dual norm of (−***β***, 1) is bounded above within the feasible region, namely,

$$\underset{\beta \in B}{\text{sup}}{\left\|\left(-\beta ,1\right)\right\|}_{*}=\bar{B}.$$

Under these two assumptions, the absolute loss could be bounded via the Cauchy-Schwarz inequality.

#### Lemma 3.1

For every feasible ***β***, it follows

$$\left|y-x^{\prime }\beta \right|\leq \bar{B}R,\;\;\text{almost}\;\text{surely.}$$

With the above result, the idea is to bound the generalization error using the empirical *Rademacher complexity* of the following class of loss functions:

$$H=\left\{(x,y)\mapsto h_{\beta }(x,y):h_{\beta }(x,y)=\left|y-x^{\prime }\beta \right|,\beta \in B\right\}.$$

We need to show that the empirical Rademacher complexity of H, denoted by $R_{N}(H)$, is upper bounded. The following result, similar to Lemma 3 in Bertsimas et al. (2015), provides a bound that is inversely proportional to the square root of the sample size.

#### Lemma 3.2


$$R_{N}(H)\leq \frac{2\bar{B}R}{\sqrt{N}}.$$

Let $\hat{\beta }$ be an optimal solution to (10), obtained using the samples (**x**_*i*_, *y*_*i*_), *i* = 1, … , *N*. Suppose we draw a new i.i.d. sample (**x**, *y*). In Theorem 3.3 we establish bounds on the error $\left|y-x^{\prime }\hat{\beta }\right|$.

#### Theorem 3.3

Under Assumptions A and B, for any 0 < *δ* < 1, with probability at least 1 − *δ* with respect to the sampling,

(14) $$E\left[\left|y-x^{\prime }\hat{\beta }\right|\right]\leq \frac{1}{N}\sum_{i=1}^{N}\left|y_{i}-x_{i}^{\prime }\hat{\beta }\right|+\frac{2\bar{B}R}{\sqrt{N}}+\bar{B}R\sqrt{\frac{8\text{log}(2/\delta )}{N}},$$

and for any $\zeta >\frac{2\bar{B}R}{\sqrt{N}}+\bar{B}R\sqrt{\frac{8\text{log}(2/\delta )}{N}}$,

(15) $$\mathbb{P}\left(\left|y-x^{\prime }\hat{\beta }\right|\geq \frac{1}{N}\sum_{i=1}^{N}\left|y_{i}-x_{i}^{\prime }\hat{\beta }\right|+\zeta \right)\leq \frac{\frac{1}{N}\sum_{i=1}^{N}\left|y_{i}-x_{i}^{\prime }\hat{\beta }\right|+\frac{2\bar{B}R}{\sqrt{N}}+\bar{B}R\sqrt{\frac{8\text{log}(2/\delta )}{N}}}{\frac{1}{N}\sum_{i=1}^{N}\left|y_{i}-x_{i}^{\prime }\hat{\beta }\right|+\zeta }.$$

There are two probability measures in the statement of Theorem 3.3. One is related to the new data (**x**, *y*), while the other is related to the samples (**x**_1_, *y*_1_), … , (**x**_*N*_, *y*_*N*_). The expectation in (14) (and the probability in (15)) is taken w.r.t. the new data (**x**, *y*). For a given set of samples, (14) (and (15)) holds with probability at least 1 − *δ* w.r.t. the measure of samples. Theorem 3.3 essentially says that given typical samples, the expected loss on new data using our Wasserstein DRO estimator could be bounded above by the average sample loss plus extra terms that depend on the supremum of ∥(−***β***, 1) ∥_*_ (our regularizer), and are proportional to $1/\sqrt{N}$. This result validates the dual norm-based regularized regression from the perspective of generalization ability, and could be generalized to any bounded loss function. It also provides implications on the form of the regularizer. For example, if given an *ℓ*_2_-loss function, the dependency on $\bar{B}$ for the generalization error bound will be of the form ${\bar{B}}^{2}$, which suggests using $\|(-\beta ,1){\|}_{*}^{2}$ as a regularizer, reducing to a variant of ridge regression (Hoerl and Kennard, 1970) for ∥ · ∥_2_ induced Wasserstein metric.

We also note that the upper bounds in (14) and (15) do not depend on the dimension of (**x**, *y*). This dimensionality-free characteristic implies direct applicability of our Wasserstein approach to high-dimensional settings and is particularly useful in many real applications where, potentially, hundreds of features may be present. Theorem 3.3 also provides guidance on the number of samples that are needed to achieve satisfactory out-of-sample performance.

#### Corollary 3.4

Suppose $\hat{\beta }$ is the optimal solution to (10). For a fixed confidence level *δ* and some threshold parameter *τ* ≥ 0, to guarantee that the percentage difference between the expected absolute loss on new data and the sample average loss is less than *τ*, that is,

$$\frac{E\left[\left|y-x^{\prime }\hat{\beta }\right|\right]-\frac{1}{N}\sum_{i=1}^{N}\left|y_{i}-x_{i}^{\prime }\hat{\beta }\right|}{\bar{B}R}\leq \tau ,$$

the sample size *N* must satisfy

(16) $$N\geq {\left[\frac{2(1+\sqrt{2\text{log}(2/\delta )})}{\tau }\right]}^{2}.$$

#### Corollary 3.5

Suppose $\hat{\beta }$ is the optimal solution to (10). For a fixed confidence level *δ*, some *τ* ∈ (0, 1) and *γ* ≥ 0, to guarantee that

$$\mathbb{P}\left(\frac{\left|y-x^{\prime }\hat{\beta }\right|-\frac{1}{N}\sum_{i=1}^{N}\left|y_{i}-x_{i}^{\prime }\hat{\beta }\right|}{\bar{B}R}\geq \gamma \right)\leq \tau ,$$

the sample size *N* must satisfy

(17) $$N\geq {\left[\frac{2(1+\sqrt{2\text{log}(2/\delta )})}{\tau \cdot \gamma +\tau -1}\right]}^{2},$$

provided that *τ* · *γ* + *τ* − 1 > 0.

In Corollaries 3.4 and 3.5, the sample size is inversely proportional to both *δ* and *τ*, which is reasonable since the more confident we want to be, the more samples we need. Moreover, the smaller *τ* is, the stricter a requirement we impose on the performance, and thus more samples are needed.

### 3.2 Discrepancy between Estimated and True Regression Planes

In addition to the generalization performance, we are also interested in the accuracy of the estimator. In this section we seek to bound the difference between the estimated and true regression coefficients, under a certain distributional assumption on (**x**, *y*). Throughout the section we will use $\hat{\beta }$ to denote the estimated regression coefficients, obtained as an optimal solution to (18), and β* for the true (unknown) regression coefficients. The bound we will derive turns out to be related to the Gaussian width (see definition in the Appendix) of the unit ball in ∥ · ∥_∞_, the sub-Gaussian norm of the uncertainty parameter (**x**, *y*), as well as the geometric structure of the true regression coefficients. We note that this proof technique may be applied to several other loss functions, e.g., *ℓ*_2_ and *ℓ*_∞_ losses, with slight modifications. However, we will see that the *ℓ*_1_-loss function incurs a relatively low estimation bias compared to others, further demonstrating the superiority of our absolute error minimization formulation.

To facilitate the analysis, we will use the following equivalent form of problem (10):

(18) $$\underset{\beta }{\text{min}}\|(-\beta ,1){\|}_{*}\;\text{s.t.}{\left\|{\left(-\beta ,1\right)}^{\prime }Z\right\|}_{1}\leq \gamma_{N},\;\beta \in B,$$

where **Z** = [(**x**_1_, *y*_1_), … , (**x**_*N*_, *y*_*N*_)] is the matrix with columns (**x**_*i*_, *y*_*i*_),*i* = 1, … , *N*, and *γ*_*N*_ is some exogenous parameter related to *ϵ*. One can show that for properly chosen *γ*_*N*_, (18) produces the same solution with (10) (Bertsekas, 1999). (18) is similar to (11) in Chen and Banerjee (2016), with the difference lying in that we impose a constraint on the error instead of the gradient, and we consider a more general notion of norm on the coefficient. On the other hand, due to their similarity, we will follow the line of development in Chen and Banerjee (2016). Still, our analysis is self-contained and the bound we obtain is in a different form, which provides meaningful insights into our specific problem. We list below the assumptions that are needed to bound the estimation error.

#### Assumption C

The *ℓ*_2_ norm of (−***β***, 1) is bounded above within the feasible region, namely,

$$\underset{\beta \in B}{\text{sup}}\|(-\beta ,1){\|}_{2}={\bar{B}}_{2}.$$

#### Assumption D (Restricted Eigenvalue Condition)

For some set $A\left(\beta^{*}\right)=\text{cone}\{v|{\left\|\left(-\beta^{*},1\right)+v\right\|}_{*}\leq {\left\|\left(-\beta^{*},1\right)\right\|}_{*}\}\cap S^{m}$ and some positive scalar *α*, where $S^{m}$ is the unit sphere in the *m*-dimensional Euclidean space,

$$\underset{v\in A\left(\beta^{*}\right)}{\text{inf}}v^{\prime }ZZ^{\prime }v\geq \underline{\alpha },$$

where $S^{m}$ denotes the unit sphere in the *m*-dimensional Euclidean space.

#### Assumption E

The true coefficient β* is a feasible solution to (18), i.e.,

$${\left\|Z^{\prime }\left(-\beta^{*},1\right)\right\|}_{1}\leq \gamma_{N},\;\beta^{*}\in B.$$

#### Assumption F

(**x**, *y*) is a centered sub-Gaussian random vector (see definition in the Appendix), i.e., it has zero mean and satisfies the following condition:

$$⦀(x,y){⦀}_{\psi_{2}}=\underset{u\in S^{m}}{\text{sup}}{⦀{\left(x,y\right)}^{\prime }u⦀}_{\psi_{2}}\leq \mu .$$

#### Assumption G

The covariance matrix of (**x**, *y*) has bounded positive eigenvalues. Set $\Gamma =E\left[(x,y){\left(x,y\right)}^{\prime }\right]$; then,

$$0<\lambda_{min}≜\lambda_{min}(\Gamma )\leq \lambda_{max}(\Gamma )≜\lambda_{max}<\infty .$$

Notice that both *α* in Assumption D and *γ*_*N*_ in Assumption E are related to the random observation matrix **Z**. A probabilistic description for these two quantities will be provided later. We next present a preliminary result, similar to Lemma 2 in Chen and Banerjee (2016), that bounds the *ℓ*_2_-norm of the estimation bias in terms of a quantity that is related to the geometric structure of the true coefficients. This result gives a rough idea on the factors that affect the estimation error, and shows the advantages of using the *ℓ*_1_-loss from the perspective of its dual norm. The bound derived in Theorem 3.6 is crude in the sense that it is a function of several random parameters that are related to the random observation matrix **Z**. This randomness will be described in a probabilistic way in the subsequent analysis.

#### Theorem 3.6

Suppose the true regression coefficient vector is β* and the solution to (18) is $\hat{\beta }$. For the set $A\left(\beta^{*}\right)=\text{cone}\left\{v|{\left\|\left(-\beta^{*},1\right)+v\right\|}_{*}\leq {\left\|\left(-\beta^{*},1\right)\right\|}_{*}\right\}\cap S^{m}$, under Assumptions A, D, and E, we have:

(19) $${\left\|\hat{\beta }-\beta^{*}\right\|}_{2}\leq \frac{2R\gamma_{N}}{\underline{\alpha }}\Psi \left(\beta^{*}\right),$$

where $\Psi \left(\beta^{*}\right)={\text{sup}}_{v\in A\left(\beta^{*}\right)}\|v{\|}_{*}$.

Notice that the bound in (19) does not explicitly depend on the sample size *N*. If we change to the *ℓ*_2_-loss function, problem (18) will become:

$$\underset{\beta }{\text{min}}\;\|(-\beta ,1){\|}_{*}\;\text{s.t.}\;{\left\|{\left(-\beta ,1\right)}^{\prime }Z\right\|}_{2}\leq \gamma_{N}\text{, }\;\beta \in B\text{.}$$

The proof of Theorem 3.6 still applies with slight modification. We will find out that in the case of *ℓ*_2_-loss, the estimation error bound takes the following form:

$${\left\|\hat{\beta }-\beta^{*}\right\|}_{2}\leq \frac{2R\sqrt{N}\gamma_{N}}{\underline{\alpha }}\Psi \left(\beta^{*}\right).$$

Similarly, the *ℓ*_∞_-loss, which considers only the maximum absolute loss among the samples, turns (18) into:

$$\underset{\beta }{\text{min}}\;\|(-\beta ,1){\|}_{*}\;\text{s.t.}\;\;{\left\|{\left(-\beta ,1\right)}^{\prime }Z\right\|}_{\infty }\leq \gamma_{N}\text{,}\;\beta \in B\text{.}$$

The corresponding bound becomes:

$${\left\|\hat{\beta }-\beta^{*}\right\|}_{2}\leq \frac{2RN\gamma_{N}}{\underline{\alpha }}\Psi \left(\beta^{*}\right).$$

We see that by using either *ℓ*_2_ or *ℓ*_∞_-loss, an explicit dependency on *N* is introduced. As a result, the estimation error bounds become worse. The reason is that for the *ℓ*_1_-loss function, its dual norm operator only picks out the maximum absolute coordinate and thus avoids the dependence on the dimension, which in our case is the sample size (see Eq.(28)), whereas other norms, e.g., *ℓ*_2_-norm, sum over all the coordinates and thus introduce a dependence on *N*.

As mentioned earlier, (19) provides a random upper bound, revealed in *α* and *γ*_*N*_, that depends on the randomness in **Z**. We therefore would like to replace these two parameters by non-random quantities. The *α* acts as the minimum eigenvalue of the matrix **ZZ**′ restricted to a subspace of ${\mathbb{R}}^{m}$, and thus a proper substitute should be related to the minimum eigenvalue of the covariance matrix of (**x**, *y*), i.e., the **Γ** matrix (cf. Assumption G), given that (**x**, *y*) is zero mean. See Lemmas 3.7, 3.8 and 3.9 for the derivation.

#### Lemma 3.7

Consider the set $A_{\Gamma }=\left\{w\in S^{m}|\Gamma^{-1/2}w\in \text{cone}\left(A\left(\beta^{*}\right)\right)\right\}$, where $A\left(\beta^{*}\right)$ is defined as in Theorem 3.6, and $\Gamma =E\left[(x,y){\left(x,y\right)}^{\prime }\right]$. Under Assumptions F and G, when the sample size $N\geq C_{1}{\bar{\mu }}^{4}{\left(w\left(A_{\Gamma }\right)\right)}^{2}$, where $\bar{\mu }=\mu \sqrt{\frac{1}{\lambda_{\text{min}}}}$, and $w\left(A_{\Gamma }\right)$ is the Gaussian width of $A_{\Gamma }$, with probability at least $1-\text{exp}\left(-C_{2}N/{\bar{\mu }}^{4}\right)$, we have

$$v^{\prime }ZZ^{\prime }v\geq \frac{N}{2}v^{\prime }\Gamma v,\;\forall v\in A\left(\beta^{*}\right),$$

where *C*_1_ and *C*_2_ are positive constants.

Note that the sample size requirement stated in Lemma 3.7 depends on the Gaussian width of $A_{\Gamma }$, where $A_{\Gamma }$ relates to $A\left(\beta^{*}\right)$. The following lemma shows that their Gaussian widths are also related. This relation is built upon the square root of the eigenvalues of **Γ**, which measures the extent to which $A_{\Gamma }$ expands $A\left(\beta^{*}\right)$.

#### Lemma 3.8 (Lemma 4 in Chen and Banerjee (2016))

Let *μ*_0_ be the *ψ*_2_-norm of a standard Gaussian random vector $g\in {\mathbb{R}}^{m}$, and $A_{\Gamma }$, $A\left(\beta^{*}\right)$ be defined as in Lemma 3.7. Then, under Assumption G,

$$w\left(A_{\Gamma }\right)\leq C_{3}\mu_{0}\sqrt{\frac{\lambda_{max}}{\lambda_{min}}}\left(w\left(A\left(\beta^{*}\right)\right)+3\right),$$

for some positive constant *C*_3_.

Combining Lemmas 3.7 and 3.8, and expressing the covariance matrix **Γ** using its eigenvalues, we arrive at the following result.

#### Corollary 3.9

Under Assumptions F and G, and the conditions in Lemmas 3.7 and 3.8, when $N\geq {\bar{C}}_{1}{\bar{\mu }}^{4}\mu_{0}^{2}\cdot \frac{\lambda_{max}}{\lambda_{min}}{\left(w\left(A\left(\beta^{*}\right)\right)+3\right)}^{2}$, with probability at least $1-\text{exp}\left(-C_{2}N/{\bar{\mu }}^{4}\right)$,

$$v^{\prime }ZZ^{\prime }v\geq \frac{N\lambda_{min}}{2},\;\forall \;v\in A\left(\beta^{*}\right),$$

where ${\bar{C}}_{1}$ and *C*_2_ are positive constants.

Next, we derive the smallest possible value of *γ*_*N*_ such that β* is feasible. The derivation uses the dual norm operator of the *ℓ*_1_-loss, resulting in a bound that depends on the Gaussian width of the unit ball in the dual norm space (∥ · ∥_∞_). See Lemma 3.10 for details.

#### Lemma 3.10

Under Assumptions C and F, for any feasible ***β***, with probability at least $1-C_{4}\text{exp}\left(-\frac{C_{5}^{2}{\left(w\left(B_{u}\right)\right)}^{2}}{4\rho^{2}}\right)$,

$${\left\|{\left(-\beta ,1\right)}^{\prime }Z\right\|}_{1}\leq C\mu {\bar{B}}_{2}w\left(B_{u}\right),$$

where $B_{u}$ is the unit ball of norm ∥·∥_∞_, $\rho ={\text{sup}}_{v\in B_{u}}\|v{\|}_{2}$, and *C*_4_, *C*_5_, *C* positive constants.

We note that for other loss functions, e.g., the *ℓ*_2_ and *ℓ*_∞_ losses, similar results can be obtained, where $B_{u}$ is defined to be the unit $\|\cdot {\|}_{*}^{\text{loss}}$-ball in ${\mathbb{R}}^{m}$, with $\|\cdot {\|}_{*}^{\text{loss}}$ being the dual norm of the loss. Combining Theorem 3.6, Corollary 3.9 and Lemma 3.10, we have the following main performance guarantee result that bounds the estimation bias of the solution to (18).

#### Theorem 3.11

Under Assumptions A, C, D, E, F, G, and the conditions of Theorem 3.6, Corollary 3.9 and Lemma 3.10, when $N\geq {\bar{C}}_{1}{\bar{\mu }}^{4}\mu_{0}^{2}\cdot \frac{\lambda_{max}}{\lambda_{min}}{\left(w\left(A\left(\beta^{*}\right)\right)+3\right)}^{2}$, with probability at least $1-\text{exp}\left(-C_{2}N/{\bar{\mu }}^{4}\right)-C_{4}\text{exp}\left(-C_{5}^{2}{\left(w\left(B_{u}\right)\right)}^{2}/\left(4\rho^{2}\right)\right)$,

(20) $${\left\|\hat{\beta }-\beta^{*}\right\|}_{2}\leq \frac{\bar{C}R{\bar{B}}_{2}\mu }{N\lambda_{min}}w\left(B_{u}\right)\Psi \left(\beta^{*}\right).$$

From (20) we see that the bias is decreased as the sample size increases and the uncertainty embedded in (**x**, *y*) (revealed in *R* and *μ*) is reduced. The estimation error bound depends on the geometric structure of the true coefficients, defined using the dual norm space of the Wasserstein metric, the Gaussian width of the unit $\|\cdot {\|}_{*}^{\text{loss}}$-ball in ${\mathbb{R}}^{m}$, and the minimum eigenvalue of the covariance matrix of (**x**, *y*), with a convergence rate 1/*N* for the *ℓ*_1_-loss we applied. As mentioned earlier, other loss functions may incur a dependence on *N* in the numerator of the bound, thus resulting in a slower convergence rate, which substantiates the benefit of using an *ℓ*_1_-loss function.

## 4. Simulation Experiments on Synthetic Datasets

In this section we will explore the robustness of the Wasserstein formulation in terms of its *Absolute Deviation (AD)* loss function and the dual norm regularizer on the *extended regression coefficient* (−***β***, 1). Recall that our Wasserstein formulation is in the following form:

(21) $$\underset{\beta \in B}{\text{inf}}\frac{1}{N}\sum_{i=1}^{N}\left|y_{i}-x_{i}^{\prime }\beta \right|+\epsilon \|(-\beta ,1){\|}_{*}.$$

We will focus on the following three aspects of this formulation:

1. How to choose a proper norm ∥ · ∥ for the Wasserstein metric?
2. Why do we penalize the extended regression coefficient (−***β***, 1) rather than ***β***?
3. What is the advantage of the AD loss compared to the *Squared Residuals (SR)* loss?

To answer Question 1, we will connect the choice of ∥ · ∥ for the Wasserstein metric with the characteristics/structures of the data (**x**, *y*). Specifically, we will design two sets of experiments, one with a dense regression coefficient β*, where all coordinates of **x** play a role in determining the value of the response *y*, and another with a sparse β* implying that only a few predictors are relevant/important in predicting *y*. Two Wasserstein formulations will be tested and compared, one induced by the ∥ · ∥_2_ (Wasserstein *ℓ*_2_), which leads to an *ℓ*_2_-regularizer in (21), and the other one induced by the ∥·∥_∞_ (Wasserstein *ℓ*_∞_) and resulting in an *ℓ*_1_-regularizer in (21). Intuitively, and based on the past experience in implementing the regularization techniques, the Wasserstein *ℓ*_2_ should outperform the Wasserstein *ℓ*_∞_ in the dense setting, while in the sparse setting, the reverse is true. Researchers have well identified the sparsity inducing property of the *ℓ*_1_-regularizer and provided a nice geometrical interpretation for it (Friedman et al., 2001). Here, we try to offer a different explanation from the perspective of the Wasserstein DRO formulation, through projecting the sparsity of β* onto the (**x**, *y*) space and establishing a *sparse* distance metric that only extracts a subset of coordinates from (**x**, *y*) to measure the closeness between samples.

For the second question, we first note that if the Wasserstein metric is induced by the following metric *s*_*c*_:

$$s_{c}(x,y)=\|(x,cy){\|}_{2},$$

for a positive constant *c*, then as *c* → ∞, the resulting Wasserstein DRO formulation becomes:

$$\underset{\beta \in B}{\text{inf}}\frac{1}{N}\sum_{i=1}^{N}\left|y_{i}-x_{i}^{\prime }\beta \right|+\epsilon \|\beta {\|}_{2},$$

which is the *ℓ*_2_-regularized LAD. This can be proved by recognizing that *s*_*c*_(**x**, *y*) = ∥(**x**, *y*)∥_**M**_, with $M\in {\mathbb{R}}^{m\times m}$ a diagonal matrix whose diagonal elements are (1, … , 1, *c*^2^), and then applying (13). Alternatively, if we let

$$s_{c}(x,y)=\|(x,cy){\|}_{\infty },$$

it can be shown that as *c* → ∞, the corresponding Wasserstein formulation becomes:

$$\underset{\beta \in B}{\text{inf}}\frac{1}{N}\sum_{i=1}^{N}\left|y_{i}-x_{i}^{\prime }\beta \right|+\epsilon \|\beta {\|}_{1},$$

which is the *ℓ*_1_-regularized LAD (see proof in the Appendix). It follows that regularizing over ***β*** implies an infinite transportation cost along *y*. In other words, for two data points (**x**_1_, *y*_1_) and (**x**_2_, *y*_2_), if *y*_1_ ≠ *y*_2_, then they are considered to be infinitely far away. By contrast, our Wasserstein formulation, which regularizes over the extended regression coefficient (−***β***, 1), stems from a finite cost along *y* that is equally weighted with **x**. We will see the disadvantages of penalizing only ***β*** in the analysis of the experimental results.

To answer Question 3, we will compare against several commonly used regression models that employ the SR loss function, e.g., ridge regression (Hoerl and Kennard, 1970), LASSO (Tibshirani, 1996), and *Elastic Net (EN)* (Zou and Hastie, 2005). We will also compare against M-estimation (Huber, 1964, 1973), which uses a variant of the SR loss and is equivalent to solving a weighted least squares problem, where the weights are determined by the residuals. These models will be compared under two different experimental setups, one involving adversarial perturbations in both **x** and *y*, and the other with perturbations only in **x**. The purpose is to investigate the behavior of these approaches when the noise in *y* is substantially reduced. As shown by Fig. 1, compared to the SR loss, the AD loss is less vulnerable to large residuals, and hence, it is advantageous in the scenarios where large perturbations appear in *y*. We are interested in studying whether its performance is consistently good when the corruptions appear mainly in **x**.

We next describe the data generation process. Each training sample has a probability *q* of being drawn from the outlying distribution, and a probability 1 − *q* of being drawn from the true (clean) distribution. Given the true regression coefficient β*, we generate the training data as follows:

- Generate a uniform random variable on [0, 1]. If it is no larger than 1 − *q*, generate a clean sample as follows:
  1. Draw the predictor $x\in {\mathbb{R}}^{m-1}$ from the normal distribution *N*_*m*−1_(**0**, **Σ**), where **Σ** is the covariance matrix of **x**, which is just the top left block of the matrix **Γ** in Assumption G. Specifically, $\Gamma =E\left[(x,y){\left(x,y\right)}^{\prime }\right]$ is equal to
$$\Gamma =\left(\begin{array}{cc} \Sigma & \Sigma \beta^{*}\\ {\left(\beta^{*}\right)}^{\prime }\Sigma & {\left(\beta^{*}\right)}^{\prime }\Sigma \beta^{*}+\sigma^{2} \end{array}\right),$$
    with *σ*^2^ being the variance of the noise term. In our implementation, **Σ** has diagonal elements equal to 1 (unit variance) and off-diagonal elements equal to *ρ*, with *ρ* the correlation between predictors.
  2. Draw the response variable *y* from *N*(**x**′***β***^∗^, *σ*^2^).
- Otherwise, depending on the experimental setup, generate an outlier that is either:
  - Abnormal in both **x** and *y*, with outlying distribution:
    1. **x** ~ *N*_*m*−1_(**0**, **Σ**) + *N*_*m*−1_(5**e**, **I**), or **x** ~ *N*_*m*−1_(**0**, **Σ**) + *N*_*m*−1_(**0**, 0.25**I**);
    2. *y* ~ *N*(**x**′***β***^∗^, *σ*^2^) + 5*σ*.
  - Abnormal only in **x**:
    1. **x** ~ *N*_*m*−1_(**0**, **Σ**) + *N*_*m*−1_(5**e**, **I**);
    2. *y* ~ *N*(**x**′***β***^∗^, *σ*^2^).
- Repeat the above procedure for *N* times, where *N* is the size of the training set.

To test the generalization ability of various formulations, we generate a test dataset containing *M* samples from the clean distribution. It is worth noting that only clean samples are included in the test set, since we only care about the prediction accuracy on clean data points, and our estimator is supposed to be consistent with the clean distribution and stay away from the outlying one. We are interested in studying the performance of various methods as the following factors are varied:

- *Signal to Noise Ratio (SNR)*, defined as:
$$\text{SNR}=\frac{{\left(\beta^{*}\right)}^{\prime }\Sigma \beta^{*}}{\sigma^{2}},$$
  which is equally spaced between 0.05 and 2 on a log scale.
- The correlation between predictors: *ρ*, which takes values in (0.1, 0.2, … , 0.9).

The performance metrics we use include:

- *Mean Squared Error (MSE)* on the test dataset, which is defined to be ${\sum_{i=1}^{M}\left(y_{i}-x_{i}^{\prime }\hat{\beta }\right)}^{2}/M$, with $\hat{\beta }$ being the estimate of β* obtained from the training set, and (**x**_*i*_, *y*_*i*_), *i* = 1, … , *M*, being the observations from the test dataset;
- *Relative Risk (RR)* of $\hat{\beta }$ defined as:
$$\text{RR}(\hat{\beta })≜\frac{{\left(\hat{\beta }-\beta^{*}\right)}^{\prime }\Sigma \left(\hat{\beta }-\beta^{*}\right)}{{\left(\beta^{*}\right)}^{\prime }\Sigma \beta^{*}}.$$
- *Relative Test Error (RTE)* of $\hat{\beta }$ defined as:
$$\text{RTE}(\hat{\beta })≜\frac{{\left(\hat{\beta }-\beta^{*}\right)}^{\prime }\Sigma \left(\hat{\beta }-\beta^{*}\right)+\sigma^{2}}{\sigma^{2}}.$$
- *Proportion of Variance Explained (PVE)* of $\hat{\beta }$ defined as:
$$\text{PVE}(\hat{\beta })≜1-\frac{{\left(\hat{\beta }-\beta^{*}\right)}^{\prime }\Sigma \left(\hat{\beta }-\beta^{*}\right)+\sigma^{2}}{{\left(\beta^{*}\right)}^{\prime }\Sigma \beta^{*}+\sigma^{2}}.$$

For the metrics that evaluate the accuracy of the estimator, i.e., the RR, RTE and PVE, we list below two types of scores, one achieved by the best possible estimator $\hat{\beta }=\beta^{*}$, called the perfect score, and the other one achieved by the null estimator $\hat{\beta }=0$, called the null score.

- RR: a perfect score is 0 and the null score is 1.
- RTE: a perfect score is 1 and the null score is SNR+1.
- PVE: a perfect score is $\frac{\text{sNR }}{\text{sNR+1 }}$, and the null score is 0.

During the training process, all the regularization parameters are tuned on a separate validation dataset. Specifically, we divide all the *N* training samples into two sets, dataset 1 and dataset 2 (validation set). For a pre-specified range of values for the penalty parameters, dataset 1 is used to train the models and derive $\hat{\beta }$, and the performance of $\hat{\beta }$ is evaluated on dataset 2. We choose the regularization parameter that yields the minimum *Median Absolute Deviation (MAD)* on the validation set. Using MAD as a selection criterion serves to hedge against the potentially large noise in the validation samples. As to the range of values for the tuned parameters, we borrow ideas from Hastie et al. (2017), where the LASSO was tuned over 50 values ranging from *λ*_*m*_ = ∥**X**′**y**∥_∞_ to a small fraction of *λ*_*m*_ on a log scale, with $X\in {\mathbb{R}}^{N\times \left(m-1\right)}$ the design matrix whose *i*-th row is $X_{i}^{\prime }$, and **y** = (*y*_1_, … , *y*_*N*_) the response vector. In our experiments, this range is properly adjusted for procedures that use the AD loss. Specifically, for Wasserstein *ℓ*_2_ and *ℓ*_∞_, *ℓ*_1_- and *ℓ*_2_-regularized LAD, the range of values for the regularization parameter is:

$$\sqrt{\text{exp}(\text{lin}\left(\text{log}\left(0.005*{\left\|X^{\prime }y\right\|}_{\infty }\right),\text{log}\left({\left\|X^{\prime }y\right\|}_{\infty }\right),50\right)),}$$

where lin(*a*, *b*, *n*) is a function that takes in scalars *a*, *b* and *n* (integer) and outputs a set of *n* values equally spaced between *a* and *b*; the exp function is applied elementwise to a vector. The square root operator is in consideration of the AD loss that is the square root of the SR loss if evaluated on a single sample.

The regularization coefficient *ϵ* in formulation (10), which is the radius of the Wasserstein ball, allows for a more efficient tuning procedure. It has been noted in Esfahani and Kuhn (2015) that for a large enough sample size, *ϵ* is inversely proportional to *N*^1/*m*^. This proportionality could be used as a guidance on setting *ϵ*, where only the proportional factor needs to be tuned (using cross-validation or a separate validation dataset as described earlier). In our implementation, given the small size of the simulated datasets, we will still adopt the validation dataset approach to tune the regularization parameter.

### 4.1 Dense β*, outliers in both x and *y*

In this subsection, we choose a dense regression coefficient β*, set the intercept $\beta_{0}^{*}=0.3$, and the coefficient for each predictor *x*_*i*_ to be $\beta_{i}^{*}=0.5$, *i* = 1, … , 20. The adversarial perturbations are present in both **x** and *y*. Specifically, the outlying distribution is described by:

1. **x** ~ *N*_*m*−1_(**0**, **Σ**) + *N*_*m*−1_(5**e**, **I**);
2. *y* ~ *N*(**x**′***β***^∗^, *σ*^2^) + 5*σ*.

**Figure 3:**
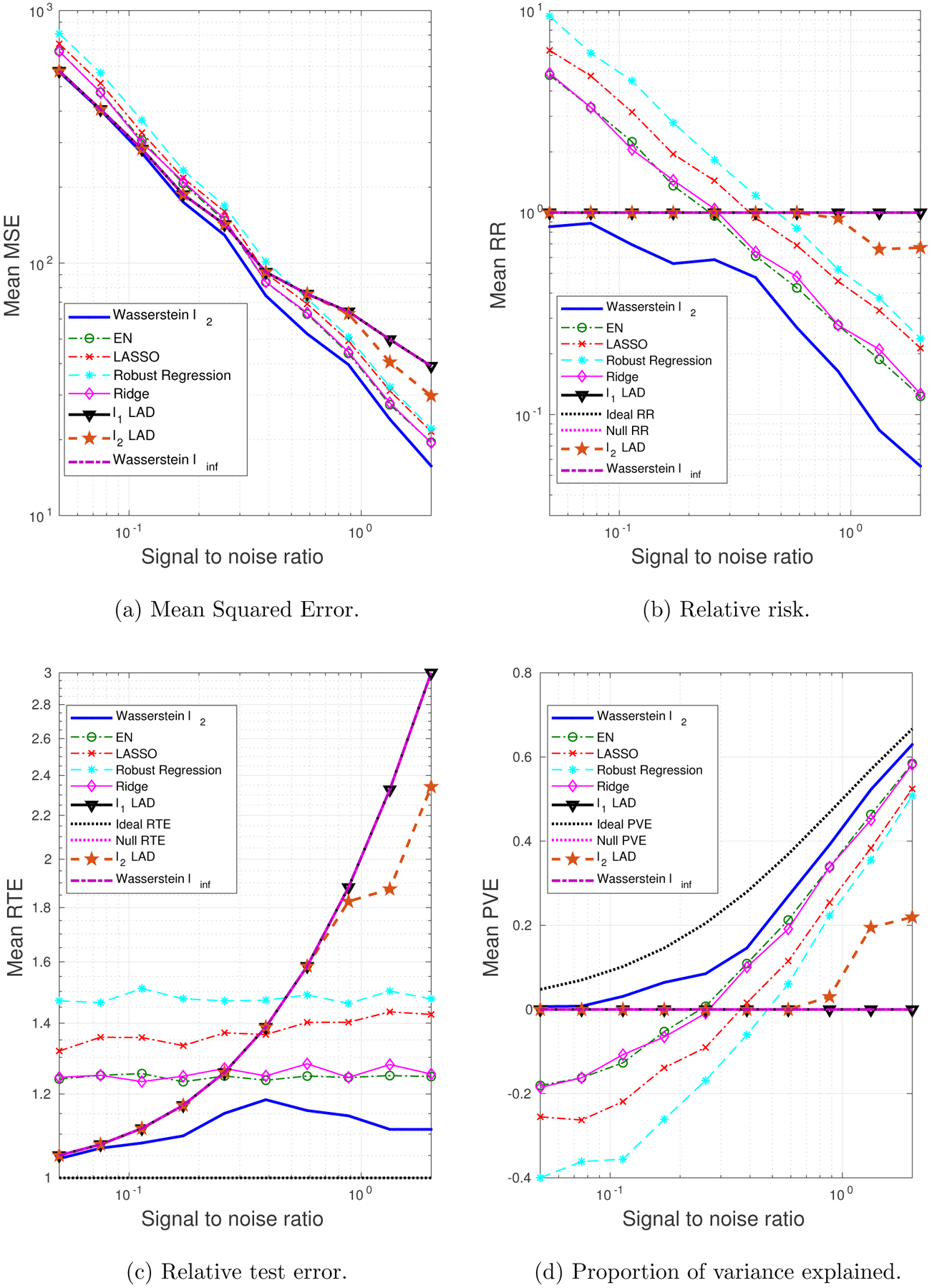
The impact of SNR on the performance metrics: dense β*, outliers in both **x** and *y*.

**Figure 4:**
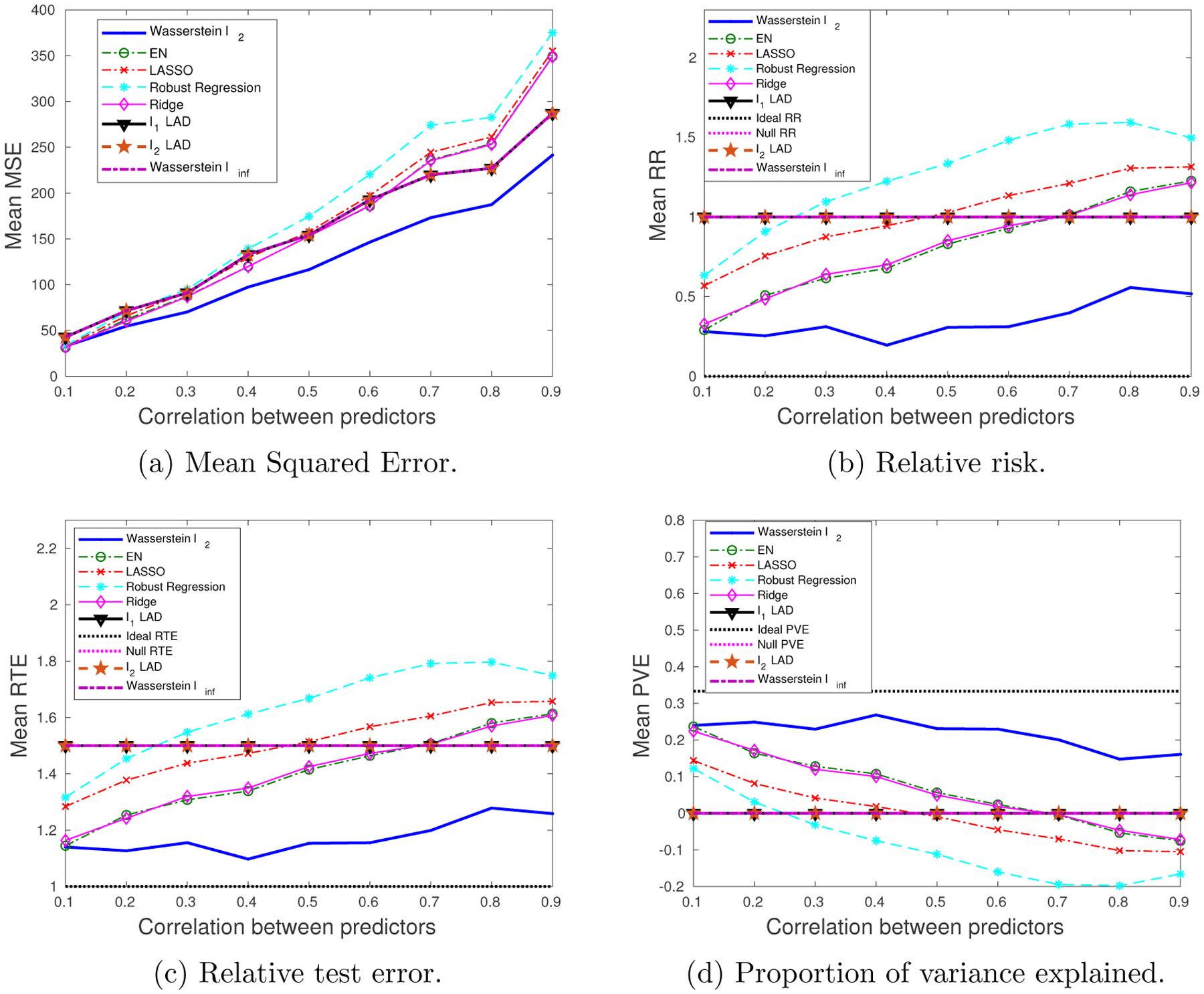
The impact of predictor correlation on the performance metrics: dense β*, outliers in both **x** and *y*.

We generate 10 datasets consisting of *N* = 100, *M* = 60 observations. The probability of a training sample being drawn from the outlying distribution is *q* = 30%. The mean values of the performance metrics (averaged over the 10 datasets), as we vary the SNR and the correlation between predictors, are shown in Figs. 3 and 4. Note that when SNR is varied, the correlation between predictors is set to 0.8 times a random noise uniformly distributed on the interval [0.2, 0.4]. When the correlation *ρ* is varied, the SNR is fixed to 0.5.

It can be seen that as the SNR decreases or the correlation between the predictors increases, the estimation problem becomes harder, and the performance of all approaches gets worse. In general the Wasserstein *ℓ*_2_ achieves the best performance in terms of all four metrics. Specifically,

- It is better than the *ℓ*_2_-regularized LAD, which assumes an infinite transportation cost along *y*.
- It is better than the Wasserstein *ℓ*_∞_ and *ℓ*_1_-regularized LAD which use the *ℓ*_1_-regularizer.
- It is better than the approaches that use the SR loss function.

Empirically we have found out that in most cases, the approaches that use the AD loss, including the *ℓ*_1_- and *ℓ*_2_-regularized LAD, and the Wasserstein *ℓ*_∞_ formulation, drive all the coordinates of ***β*** to zero, due to the relatively small magnitude of the AD loss compared to the norm of the coefficient, so that the regularizer dominates the solution. The approaches that use the SR loss, e.g., ridge regression and EN, do not exhibit such a problem, since the squared residuals weaken the dominance of the regularization term.

Overall the *ℓ*_2_-regularizer outperforms the *ℓ*_1_-regularizer, since the true regression coefficient is dense, which implies that a proper distance metric on the (**x**, *y*) space should take into account all the coordinates. From the perspective of the Wasserstein DRO framework, the *ℓ*_1_-regularizer corresponds to an ∥ · ∥_∞_-based distance metric on the (**x**, *y*) space that only picks out the most influential coordinate to determine the closeness between data points, which in our case is not reasonable since every coordinate plays a role (reflected in the dense β*). In contrast, if β* is sparse, using the ∥ · ∥_∞_ as a distance metric on (**x**, *y*) is more appropriate. A more detailed discussion of this will be presented in Sections 4.3 and 4.4.

### 4.2 Dense *β*^∗^, outliers only in x

In this subsection we will experiment with the same β* as in Section 4.1, but with perturbations only in **x**, i.e., for a given **x** of the outlier, the corresponding *y* value is drawn in the same way as the clean samples. Our goal is to investigate the performance of the Wasserstein formulation when the response *y* is not subjected to large perturbations. The motivation for introducing the AD loss in the Wasserstein formulation is to hedge against large residuals, as illustrated in Fig. 1. We are interested in comparing the AD and SR loss functions when the residuals have moderate magnitudes.

**Figure 5:**
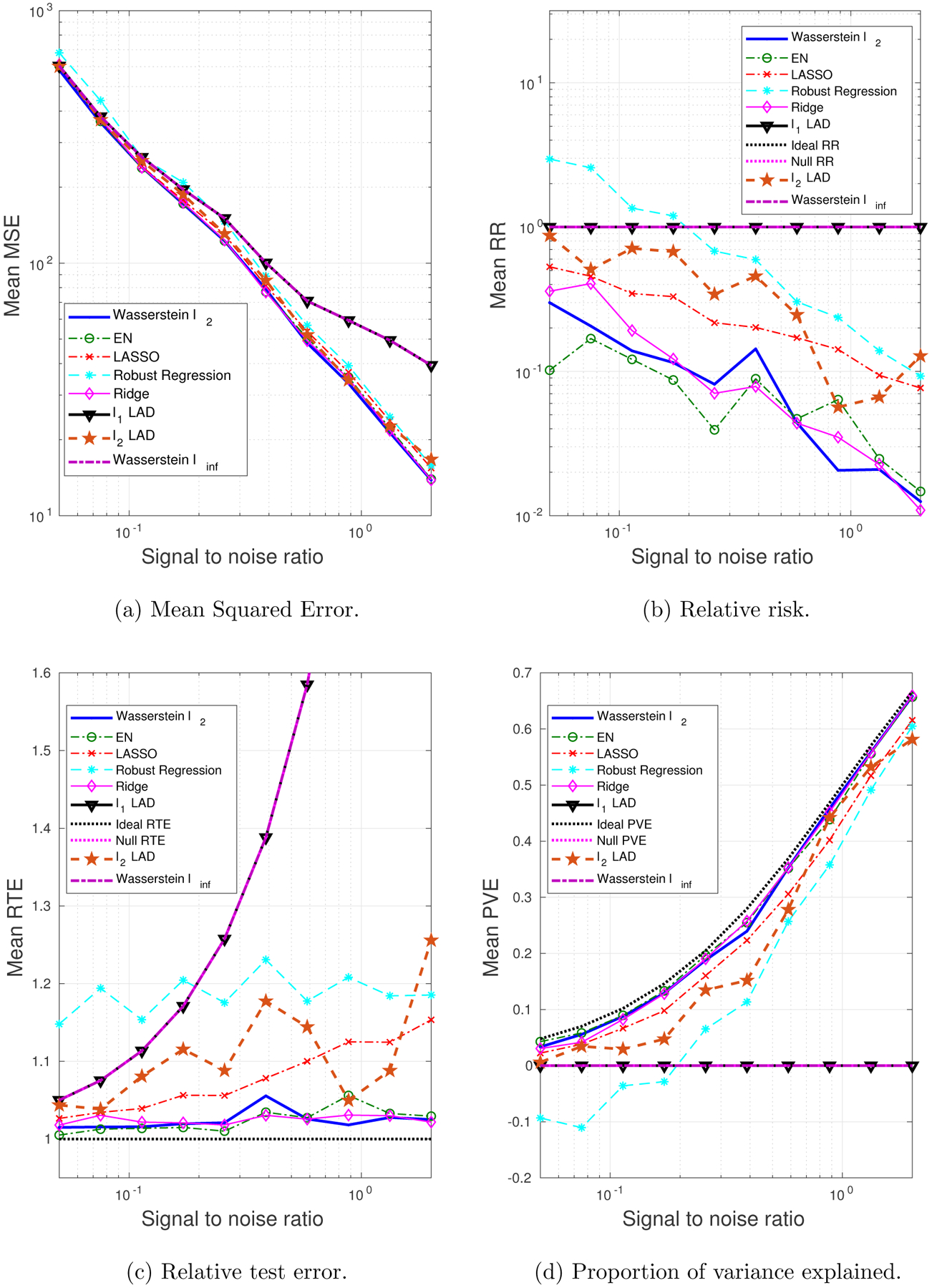
The impact of SNR on the performance metrics: dense β*, outliers only in **x**.

**Figure 6:**
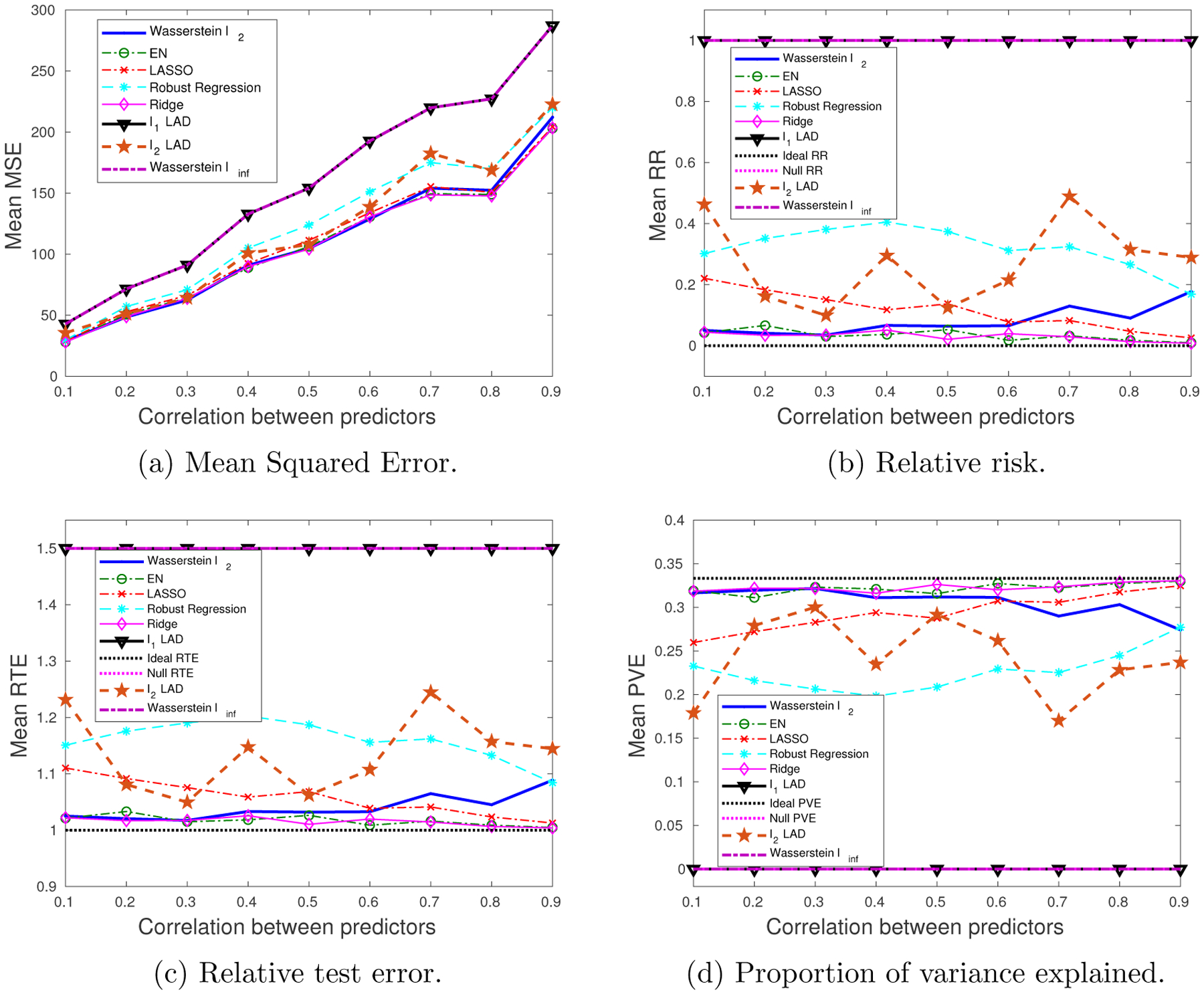
The impact of predictor correlation on the performance metrics: dense β*, outliers only in **x**.

Interestingly, we have observed that although the *ℓ*_1_- and *ℓ*_2_-regularized LAD, as well as the Wasserstein *ℓ*_∞_ formulation, exhibit unsatisfactory performance, the Wasserstein *ℓ*_2_, which shares the same loss function with them, is able to achieve a comparable performance with the best among all – EN and ridge regression (see Figs. 5 and 6). Notably, the *ℓ*_2_-regularized LAD, which is just slightly different from our Wasserstein *ℓ*_2_ formulation, shows a much worse performance. This is because the *ℓ*_2_-regularized LAD implicitly assumes an infinite transportation cost along *y*, which gives zero tolerance to the variation in the response. For example, given two data points (**x**_1_, *y*_1_) and (**x**_2_, *y*_2_), as long as *y*_1_ ≠ *y*_2_, the distance between them is infinity. Therefore, a reasonable amount of fluctuation, caused by the intrinsic randomness of *y*, would be overly exaggerated by the underlying metric used by the *ℓ*_2_-regularized LAD. In contrast, our Wasserstein approach uses a proper notion of norm to evaluate the distance in the (**x**, *y*) space and is able to effectively distinguish abnormally high variations from moderate, acceptable noise.

It is also worth noting that the formulations with the AD loss, e.g., *ℓ*_2_- and *ℓ*_1_-regularized LAD, and the Wasserstein *ℓ*_∞_, perform worse than the approaches with the SR loss. One reasonable explanation is that the AD loss, introduced primarily for hedging against large perturbations in *y*, is less useful when the noise in *y* is moderate, in which case the sensitivity to response noise is needed. Although the AD loss is not a wise choice, penalizing the extended coefficient vector (−***β***, 1) seems to make up, making the Wasserstein *ℓ*_2_ a competitive method even when the perturbations appear only in **x**.

### 4.3 Sparse β*, outliers in both x and *y*

In this subsection we will experiment with a sparse β*. The intercept is set to $\beta_{0}^{*}=3$, and the coefficients for the 20 predictors are set to β* = (0.05, 0,0.006, 0, −0.007, 0, 0.008, 0, … , 0). The adversarial perturbations are present in both **x** and *y*. Specifically, the distribution of outliers is characterized by:

1. **x** ~ *N*_*m*−1_(**0**, **Σ**) + *N*_*m*−1_(**0**, 0.25**I**);
2. *y* ~ *N*(**x**′β*, *σ*^2^) + 5*σ*.

**Figure 7:**
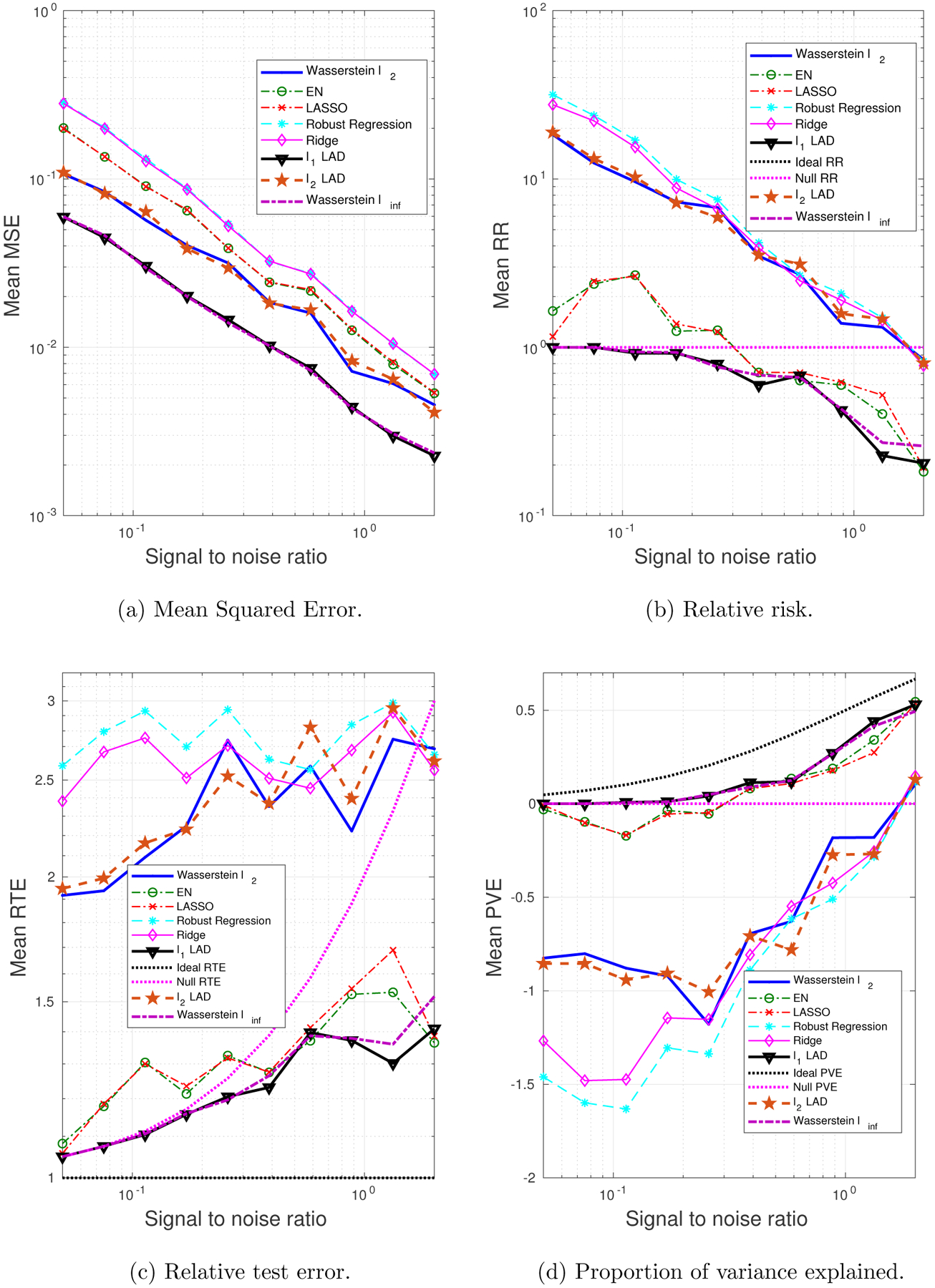
The impact of SNR on the performance metrics: sparse β*, outliers in both **x** and *y*.

**Figure 8:**
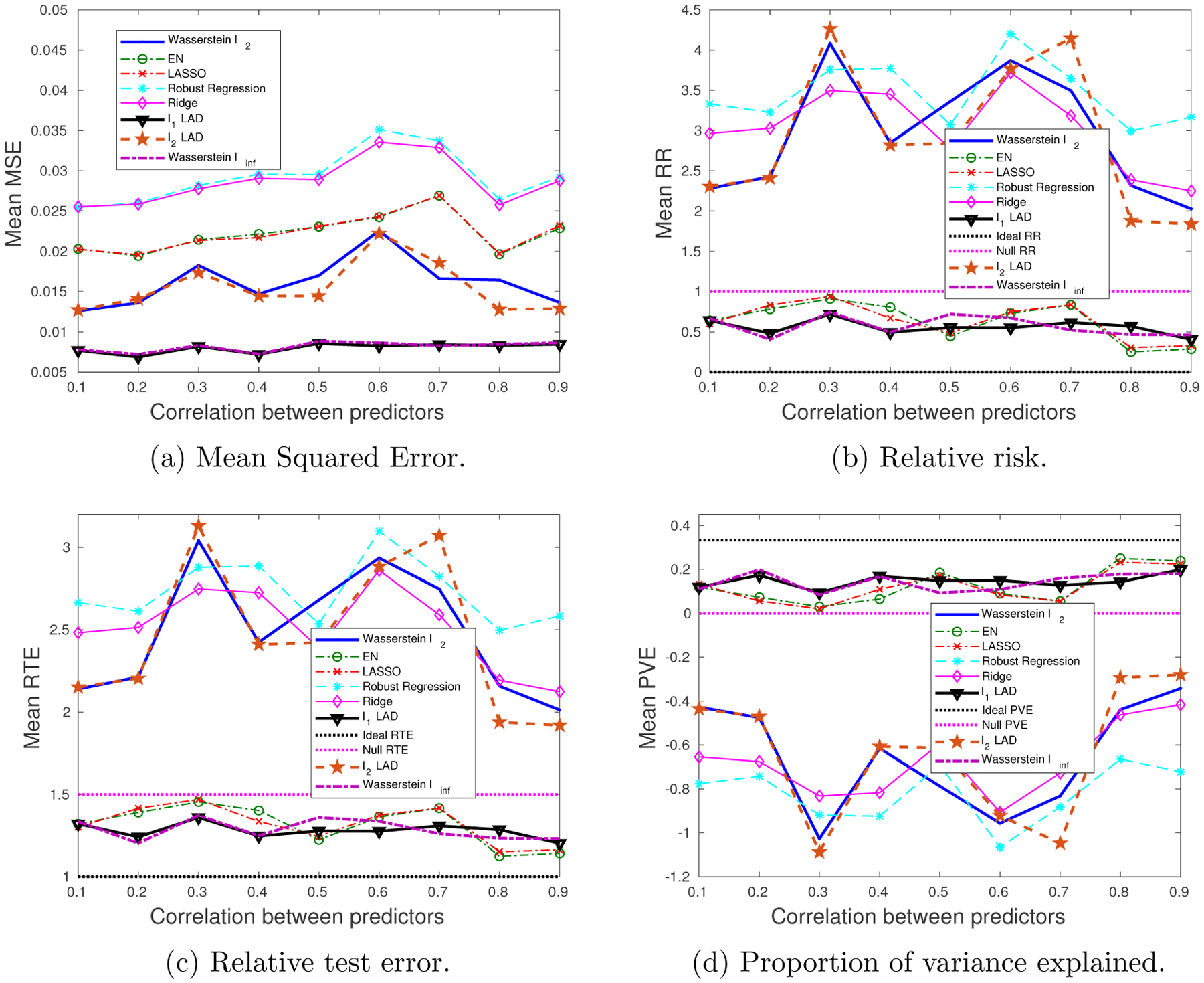
The impact of predictor correlation on the performance metrics: sparse β*, outliers in both **x** and *y*.

Our goal is to study the impact of the sparsity of β* on the choice of the norm space for the Wasserstein metric. We know that the *ℓ*_1_-regularizer works better than the *ℓ*_2_-regularizer for sparse data, which has been validated by our results in Figs. 7 and 8. We will see that the Wasserstein *ℓ*_∞_ formulation significantly outperforms the Wasserstein *ℓ*_2_. An intuitively appealing interpretation for the sparsity inducing property of the *ℓ*_1_-regularizer is made available by the Wasserstein DRO framework, which we explain as follows. The sparse regression coefficient β* implies that only a few predictors are relevant to the regression model, and thus when measuring the distance in the (**x**, *y*) space, we need a metric that only extracts the subset of relevant predictors. The ‖ · ‖_∞_, which takes only the most influential coordinate of its argument, roughly serves this purpose. Compared to the ‖ · ‖_2_ which takes into account all the coordinates, most of which are redundant due to the sparsity assumption, ‖ · ‖_∞_ results in a better performance, and hence, the Wasserstein *ℓ*_∞_ formulation that stems from the ‖ · ‖_∞_ distance metric on (**x**, *y*) and induces the *ℓ*_1_-regularizer is expected to outperform others.

We note that the *ℓ*_1_-regularized LAD achieves similar performance to ours, since replacing ‖***β***‖_1_ by ‖(−***β***, 1)‖_1_ only adds a constant term to the objective function. The generalization performance (mean MSE) of the AD loss-based formulations is consistently better than those with the SR loss, since the AD loss is less affected by large perturbations in *y*. Also note that choosing a wrong norm for the Wasserstein metric, e.g., the Wasserstein *ℓ*_2_, could lead to an enormous estimation error, whereas with a right norm space, we are guaranteed to outperform all others. Even when the SNR is very low, our performance is at least as good as the null estimator (see Fig. 7). Although EN and LASSO achieve similar performance to ours for moderate SNR values, they have a chance of performing even worse than the null estimator when there is little signal/information to learn from.

### 4.4 Sparse β*, outliers only in x

In this subsection, we will use the same sparse coefficient as in Section 4.3, but the perturbations are present only in **x**. Specifically, for outliers, their predictors and responses are drawn from the following distributions:

1. **x** ~ *N*_*m*−1_(**0**, **Σ**) + *N*_*m*−1_(5**e**, **I**);
2. *y* ~ *N*(**x**′β*, *σ*^2^).

**Figure 9:**
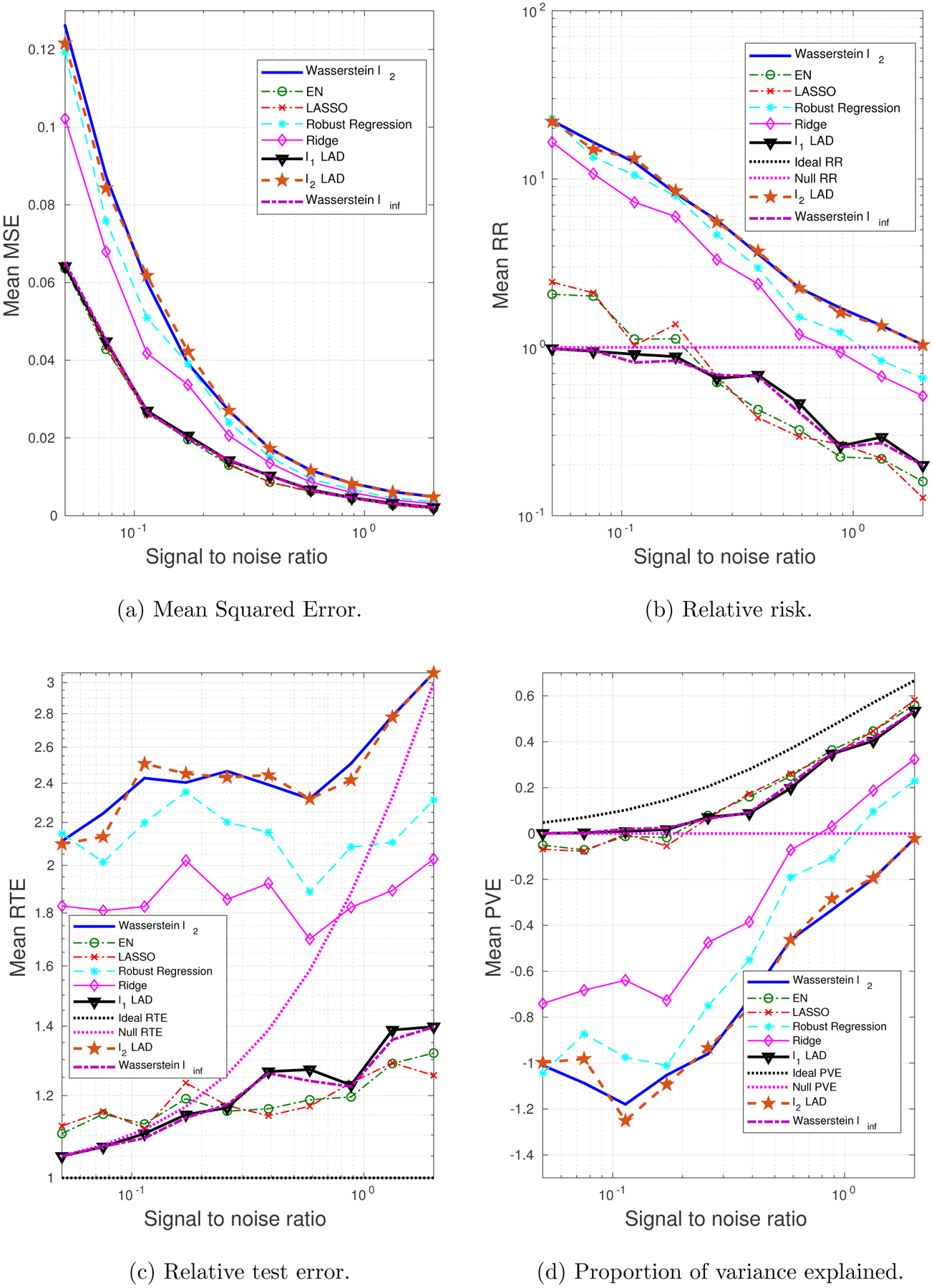
The impact of SNR on the performance metrics: sparse β*, outliers only in **x**.

Not surprisingly, the Wasserstein *ℓ*_∞_ and the *ℓ*_1_-regularized LAD achieve the best performance. Notice that in Section 4.3, where perturbations appear in both **x** and *y*, the AD loss-based formulations have smaller generalization and estimation errors than the SR loss-based formulations. When we reduce the variation in *y*, the SR loss seems superior to the AD loss, if we restrict attention to the improperly regularized (*ℓ*_2_-regularizer) formulations (see Fig. 9). For the *ℓ*_1_-regularized formulations, our Wasserstein *ℓ*_∞_ formulation, as well as the *ℓ*_1_-regularized LAD, is comparable with the EN and LASSO. Moreover, when there is little information to utilize (low SNR), EN and LASSO are worse than the null estimator, whereas our performance is at least as good as the null estimator.

We summarize below our main findings from all sets of experiments we have presented:

1. When a proper norm space is selected for the Wasserstein metric, the Wasserstein DRO formulation outperforms all others in terms of the generalization and estimation qualities.
2. Penalizing the extended regression coefficient (−***β***, 1) implicitly assumes a more reasonable distance metric on (**x**, *y*) and thus leads to a better performance.
3. The AD loss is remarkably superior to the SR loss when there is large variation in the response *y*.
4. The Wasserstein DRO formulation shows a more stable estimation performance than others when the correlation between predictors is varied.

### 4.5 An outlier detection example

As an application, we consider an unlabeled two-class classification problem, where our goal is to identify the abnormal class of data points based on the predictor and response information using the Wasserstein formulation. We do not know a priori whether the samples are normal or abnormal, and thus classification models do not apply. The commonly used regression model for this type of problem is the M-estimation (Huber, 1964, 1973), against which we will compare in terms of the outlier detection capability.

The data are generated in the same fashion as before. For clean samples, all predictors *x*_1_, … , *x*_30_ come from a normal distribution with mean 7.5 and standard deviation 4.0. The response is a linear function of the predictors with $\beta_{0}^{*}=0.3$, $\beta_{1}^{*}=\cdots =\beta_{30}^{*}=0.5$, plus a Gaussian distributed noise term with zero mean and standard deviation *σ*. The outliers concentrate in a cloud that is randomly placed in the interior of the **x**-space. Specifically, their predictors are uniformly distributed on (*u* − 0.125, *u* + 0.125), where *u* is a uniform random variable on (7.5 − 3 × 4, 7.5 + 3 × 4). The response values of the outliers are at a *δ*_*R*_ distance off the regression plane.

$$y=\beta_{0}^{*}+\beta_{1}^{*}x_{1}+\cdots +\beta_{30}^{*}x_{30}+\delta_{R}.$$

We will compare the performance of the Wasserstein *ℓ*_2_ formulation (10) with the *ℓ*_1_-regularized LAD and M-estimation with three cost functions – Huber (Huber, 1964, 1973), Talwar (Hinich and Talwar, 1975), and Fair (Fair, 1974). The performance metrics include the *Receiver Operating Characteristic (ROC)* curve which plots the true positive rate against the false positive rate, and the related *Area Under Curve (AUC)*.

Notice that all the regression methods under consideration only generate an estimated regression coefficient. The identification of outliers is based on the residual and estimated standard deviation of the noise. Specifically,

$$\text{ Outlier }=\{\begin{array}{ll} \text{YES}, & \text{ if }|\text{residual}|>\text{ threshold }\times \hat{\sigma },\\ \text{NO}, & \text{ otherwise, } \end{array}$$

where $\hat{\sigma }$ is the standard deviation of residuals in the entire training set. ROC curves are obtained through adjusting the threshold value.

**Figure 11:**
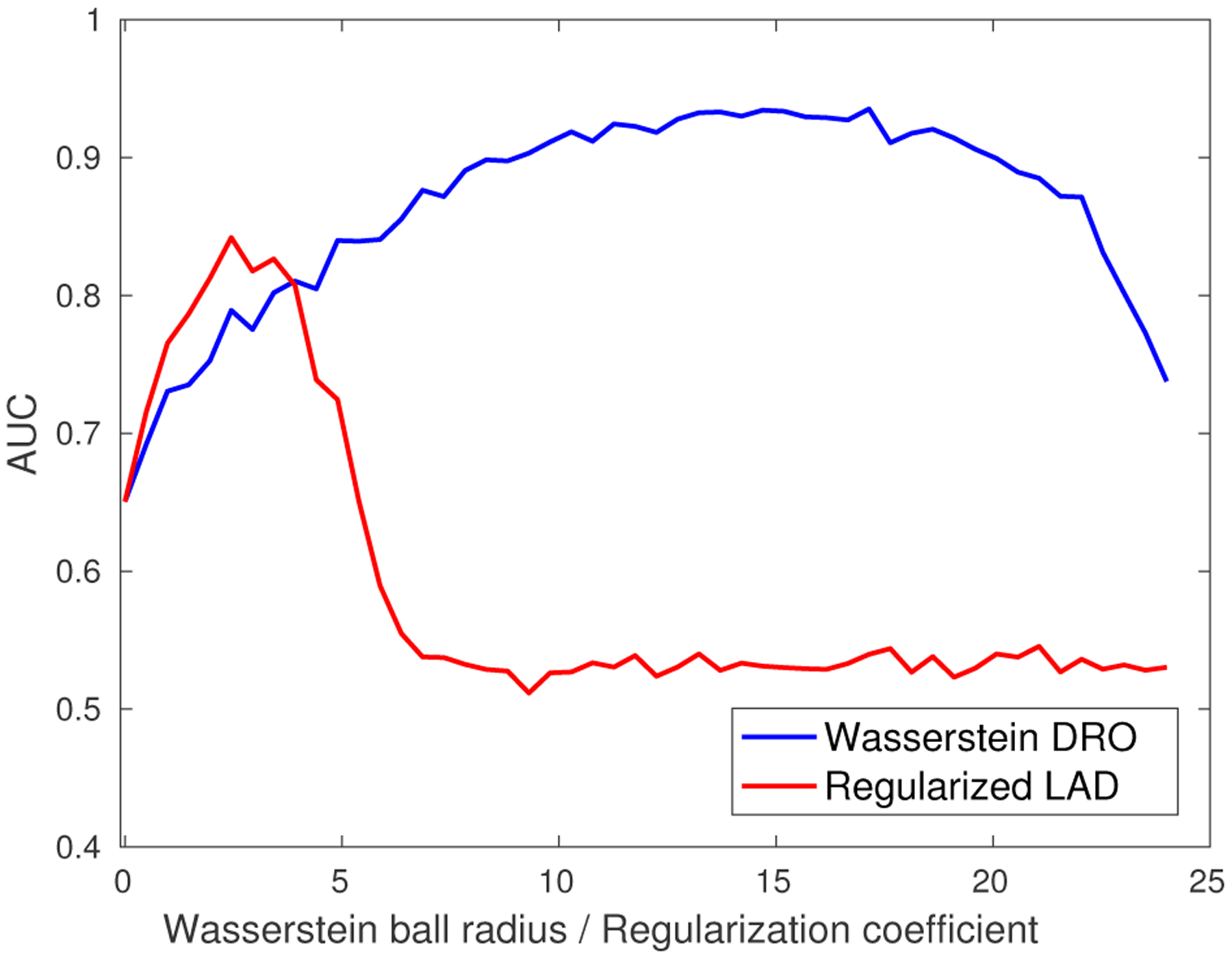
Out-of-sample AUC v.s. Wasserstein ball radius (regularization coefficient).

The regularization parameters for Wasserstein DRO and regularized LAD are tuned using a separate validation set as done in previous sections. We would like to highlight a salient advantage of our approach reflected in its robustness w.r.t. the choice of *ϵ*. In Fig. 11 we plot the out-of-sample AUC as the radius *ϵ* (regularization parameter) varies, for the *ℓ*_2_-induced Wasserstein DRO and the *ℓ*_1_-regularized LAD. For the Wasserstein DRO curve, when *ϵ* is small, the Wasserstein ball contains the true distribution with low confidence and thus AUC is low. On the other hand, too large *ϵ* makes our solution overly conservative. Note that the robustness of our approach, indicated by the flatness of the Wasserstein DRO curve, constitutes another advantage, whereas the performance of LAD dramatically deteriorates once the regularizer deviates from the optimum. Moreover, the maximal achievable AUC for Wasserstein DRO is significantly higher than LAD.

**Figure 12:**
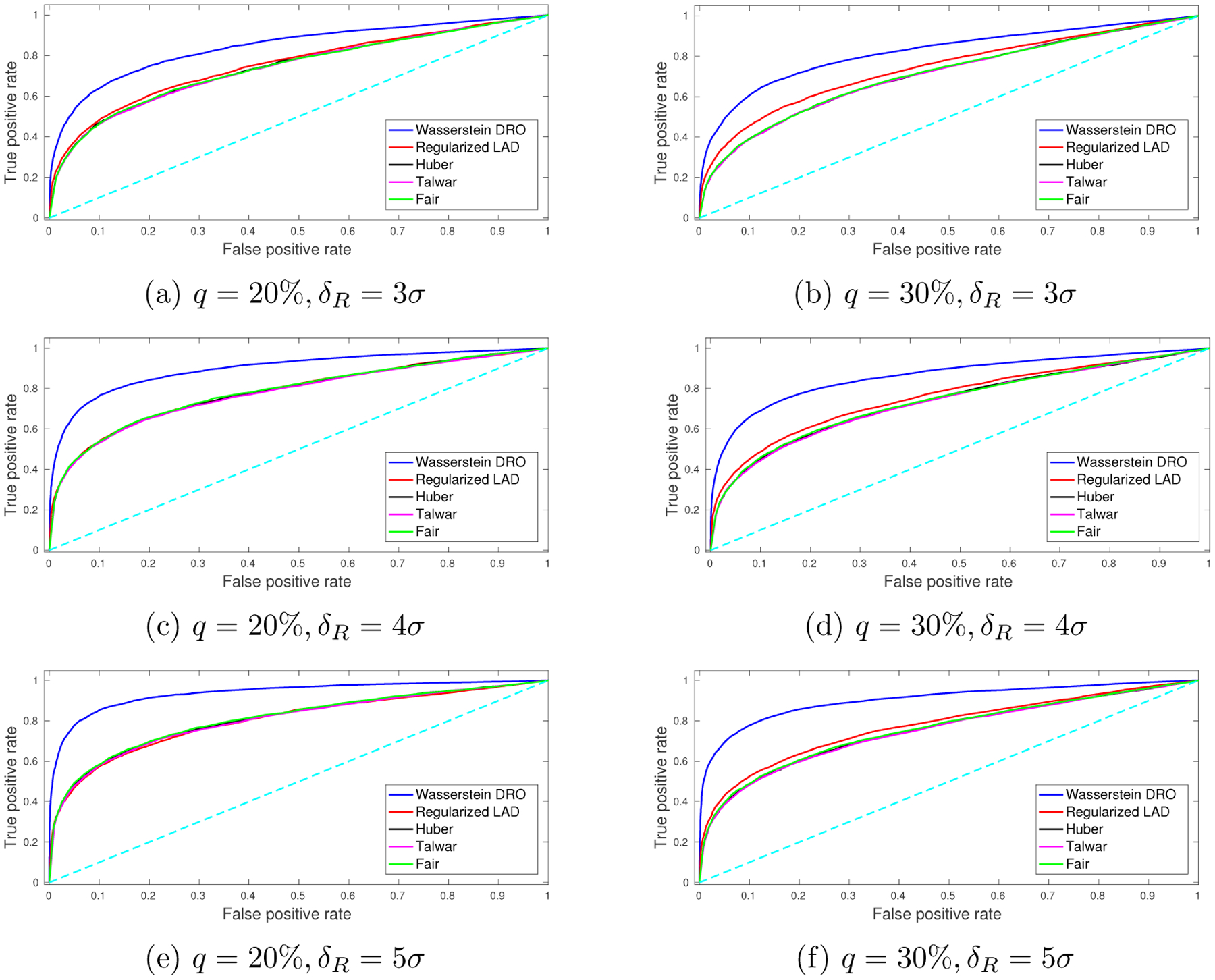
ROC curves for outliers in a randomly placed cloud, *N* = 60, *σ* = 0.5.

In Fig. 12 we show the ROC curves for different approaches, where *q* represents the percentage of outliers, and *δ*_*R*_ the outlying distance along *y*. We see that the Wasserstein DRO formulation consistently outperforms all other approaches, with its ROC curve lying well above others. In general, all approaches have better performance when the percentage of outliers is lower, and the outlying distance is larger. The approaches that use the AD loss function (e.g., Wasserstein DRO and regularized LAD) tend to outperform those that adopt the SR loss (e.g., M-estimation which uses a variant of the SR loss). The superiority of our formulation could be attributed to the AD loss function, and the distributional robustness since we hedge against a family of plausible distributions, including the true distribution with high confidence. By contrast, M-estimation adopts an *Iteratively Reweighted Least Squares (IRLS)* procedure which assigns weights to data points based on the residuals from previous iterations, and then solves a weighted least squares estimation problem. With such an approach, there is a chance of exaggerating the influence of outliers while downplaying the importance of clean observations, especially when the initial residuals are obtained through *Ordinary Least Squares (OLS)*.

## 5. Conclusions

We presented a novel *ℓ*_1_-loss based robust learning procedure using *Distributionally Robust Optimization (DRO)* in a linear regression framework, through which a delicate connection between the metric space on data and the regularization term has been established. The Wasserstein metric was utilized to construct the ambiguity set and a tractable reformulation was derived. It is worth noting that the linear law assumption does not necessarily limit the applicability of our model. In fact, by appropriately pre-processing the data, one can often find a roughly linear relationship between the response and transformed explanatory variables. Our Wasserstein formulation incorporates a class of models whose specific form depends on the norm space that the Wasserstein metric is defined on. We provide out-of-sample generalization guarantees, and bound the estimation bias of the general formulation. Extensive numerical examples demonstrate the superiority of the Wasserstein formulation and shed light on the advantages of the *ℓ*_1_-loss, the implication of the regularizer, and the selection of the norm space for the Wasserstein metric. We also presented an outlier detection example as an application of this robust learning procedure. A remarkable advantage of our approach rests in its flexibility to adjust the form of the regularizer based on the characteristics of the data.

## Figures and Tables

**Figure 10:**
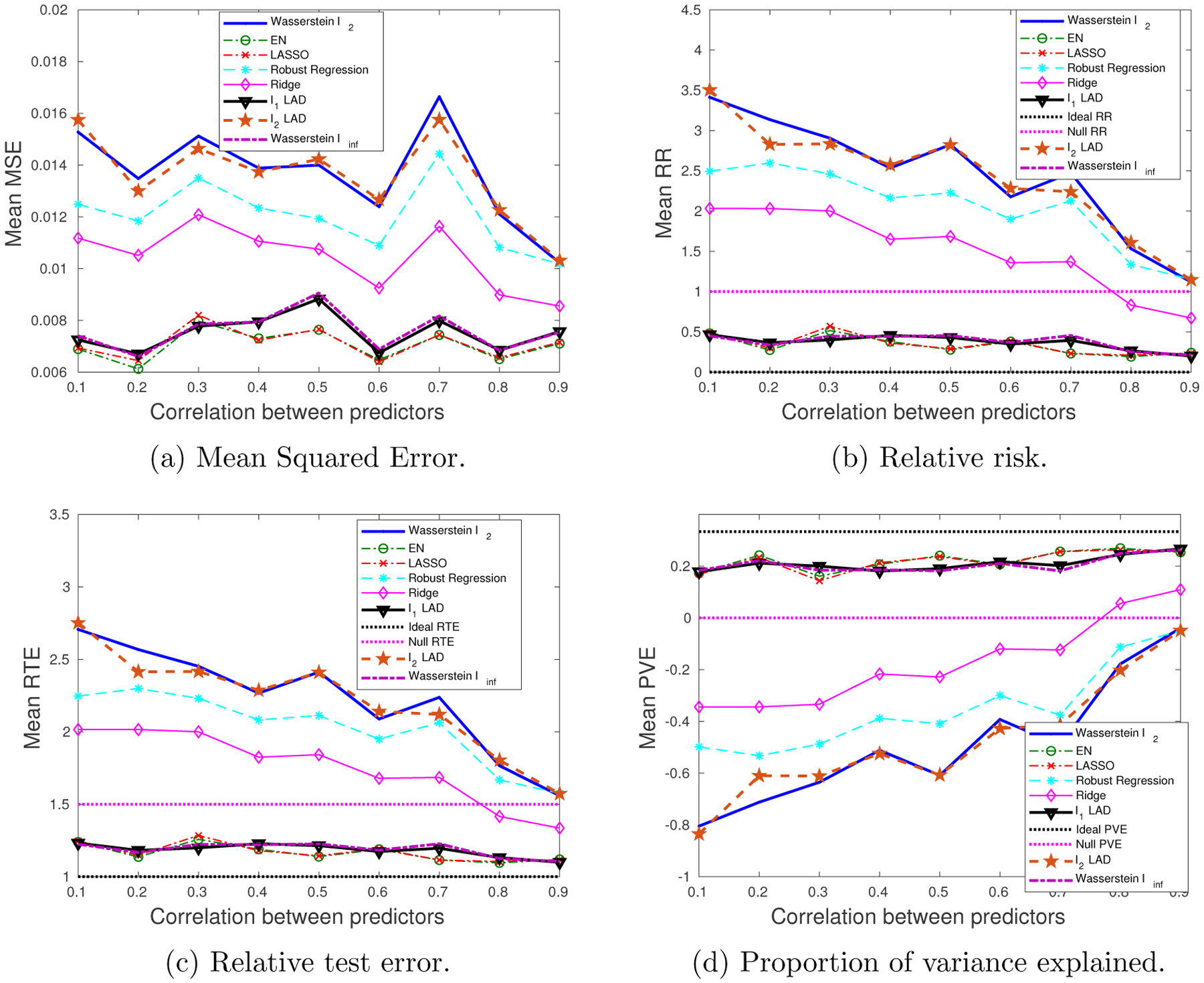
The impact of predictor correlation on the performance metrics: sparse β*, outliers only in **x**.

## Appendix A. Omitted Definitions and Proofs

This section includes proofs for the theorems and lemmas, in the order they appear in the paper.

### A.1 Proof of Theorem 2.1

#### Proof

We will adopt the notation $z≜(x,y),\tilde{\beta }≜(-\beta ,1)$ for ease of analysis. First rewrite *κ*(***β***) as:

$$\kappa (\beta )=\text{sup}\left\{\|\theta {\|}_{*}:\underset{z|z^{\prime }\tilde{\beta }\geq 0}{\text{sup}}\left\{{\left(\theta -\tilde{\beta }\right)}^{\prime }z\right\}<\infty ,\underset{z|z^{\prime }\beta \leq 0}{\text{sup}}\left\{{\left(\theta +\tilde{\beta }\right)}^{\prime }z\right\}<\infty \right\}.$$

Consider now the two linear optimization problems A and B:

$$\text{Problem A:}\;\begin{array}{c} \text{max}\;{\left(\theta -\tilde{\beta }\right)}^{\prime }z\\ \text{s.t.}\;z^{\prime }\tilde{\beta }\geq 0. \end{array}$$

$$\text{Problem B:}\;\begin{array}{c} \text{max}\;{\left(\theta +\tilde{\beta }\right)}^{\prime }z\\ \text{s.t.}\;z^{\prime }\tilde{\beta }\leq 0. \end{array}$$

Form the dual problems using dual variables *r*_*A*_ and *r*_*B*_, respectively:

$$\text{Dual-A:}\;\begin{array}{l} \text{min}\;0\cdot r_{A}\\ \text{s.t.}\underset{r_{A}\leq 0,}{\;\tilde{\beta }r_{A}=\theta -\tilde{\beta }}, \end{array}$$

$$\text{Dual-B:}\;\begin{array}{l} \text{min}\;0\cdot r_{B}\\ \text{s}\underset{r_{B}\leq 0.}{\text{.t.}\;\tilde{\beta }r_{B}=\theta -\tilde{\beta }}, \end{array}$$

We want to find the set of ***θ*** such that the optimal values of problems *A* and *B* are finite. Then, Dual-A and Dual-B need to have non-empty feasible sets, which implies the following two conditions:

(22)
$$\exists r_{A}\leq 0,\;\text{s.t.}\;\tilde{\beta }r_{A}=\theta -\tilde{\beta },$$

(23)
$$\exists r_{B}\geq 0,\;\text{s.t.}\;\tilde{\beta }r_{B}=\theta +\tilde{\beta }\text{.}$$

For all *i* with ${\tilde{\beta }}_{i}\leq 0$, (22) implies $\theta_{i}-{\tilde{\beta }}_{i}\geq 0$ and (23) implies $\theta_{i}\leq -{\tilde{\beta }}_{i}$. On the other hand, for all *j* with ${\tilde{\beta }}_{j}\geq 0$, (22) and (23) imply $-{\tilde{\beta }}_{j}\leq \theta_{j}\leq {\tilde{\beta }}_{j}$. It is not hard to conclude that:

$$\left|\theta_{i}\right|\leq \left|{\tilde{\beta }}_{i}\right|,\;\forall i.$$

It follows,

$$\kappa (\beta )=\text{sup}\left\{\|\theta {\|}_{*}:\left|\theta_{i}\right|\leq \left|{\tilde{\beta }}_{i}\right|,\forall i\right\}=\|\tilde{\beta }{\|}_{*}\text{.}$$

### A.2 Proof of Lemma 3.2

#### Proof

Suppose that *σ*_1_, … , *σ*_*N*_ are i.i.d. uniform random variables on {1, −1}. Then, by the definition of the Rademacher complexity and Lemma 3.1,

$$R_{N}(H)=E\left[\underset{h\in H}{\text{sup}}\frac{2}{N}\left|\sum_{i=1}^{N}\sigma_{i}h_{\beta }\left(x_{i},y_{i}\right)\right||\left(x_{1},y_{1}\right),\ldots ,\left(x_{N},y_{N}\right)\right]\;\leq \frac{2\bar{B}R}{N}E\left[\left|\sum_{i=1}^{N}\sigma_{i}\right|\right]\;\leq \frac{2\bar{B}R}{N}E\left[\sqrt{\sum_{i=1}^{N}\sigma_{i}^{2}}\right]\;=\frac{2\bar{B}R}{\sqrt{N}}.$$

### A.3 Proof of Theorem 3.3

#### Proof

We use Theorem 8 in Bartlett and Mendelson (2002), setting the following correspondences with the notation used there: $L(x,y)=\phi (x,y)=\left|y-x^{\prime }\beta \right|$. This yields the bound (14) on the expected loss. For Eq. (15), we apply Markov’s inequality to obtain:

$$\mathbb{P}\left(\left|y-x^{\prime }\hat{\beta }\right|\geq \frac{1}{N}\sum_{i=1}^{N}\left|y_{i}-x_{i}^{\prime }\hat{\beta }\right|+\zeta \right)\leq \frac{E\left[\left|y-x^{\prime }\hat{\beta }\right|\right]}{\frac{1}{N}\sum_{i=1}^{N}\left|y_{i}-x_{i}^{\prime }\hat{\beta }\right|+\zeta }\;\leq \frac{\frac{1}{N}\sum_{i=1}^{N}\left|y_{i}-x_{i}^{\prime }\hat{\beta }\right|+\frac{2\bar{B}R}{\sqrt{N}}+\bar{B}R\sqrt{\frac{8\text{log}(2/\delta )}{N}}}{\frac{1}{N}\sum_{i=1}^{N}\left|y_{i}-x_{i}^{\prime }\hat{\beta }\right|+\zeta }.$$

### A.4 Proof of Corollary 3.4

#### Proof

The percentage difference requirement can be translated into:

$$\frac{2}{\sqrt{N}}+\sqrt{\frac{8\text{log}(2/\delta )}{N}}\leq \tau ,$$

from which (16) can be easily derived. ■

### A.5 Proof of Corollary 3.5

#### Proof

Based on Theorem 3.3, we just need the following inequality to hold:

$$\frac{\frac{1}{N}\sum_{i=1}^{N}\left|y_{i}-x_{i}^{\prime }\hat{\beta }\right|+\frac{2\bar{B}R}{\sqrt{N}}+\bar{B}R\sqrt{\frac{8\text{log}(2/\delta )}{N}}}{\frac{1}{N}\sum_{i=1}^{N}\left|y_{i}-x_{i}^{\prime }\hat{\beta }\right|+\gamma \bar{B}R}\leq \tau ,$$

which is equivalent to:

(24)
$$\frac{\gamma \bar{B}R-\frac{2\bar{B}R}{\sqrt{N}}-\bar{B}R\sqrt{\frac{8\text{log}(2/\delta )}{N}}}{\frac{1}{N}\sum_{i=1}^{N}\left|y_{i}-x_{i}^{\prime }\hat{\beta }\right|+\gamma \bar{B}R}\geq 1-\tau .$$

We cannot obtain a lower bound for *N* by directly solving (24) since *N* appears in a summation operator. A proper relaxation to (24) is:

(25)
$$\frac{\gamma -\frac{2}{\sqrt{N}}-\sqrt{\frac{8\text{log}(2/\delta )}{N}}}{1+\gamma }\geq 1-\tau ,$$

due to the fact that $\frac{1}{N}\sum_{i=1}^{N}\left|y_{i}-x_{i}^{\prime }\hat{\beta }\right|\leq \bar{B}R$. By solving (25), we obtain (17). ■

### A.6 Sub-Gaussian Random Variables and Gaussian Width

#### Definition 1 (Sub-Gaussian random variable)

A random variable *z* is sub-Gaussian if the *ψ*_2_-norm defined below is finite, i.e.,

$$⦀z{⦀}_{\psi_{2}}≜\underset{q\geq 1}{\text{sup}}\frac{E|z{|}^{q}}{\sqrt{q}}<+\infty .$$

An equivalent property for sub-Gaussian random variables is that their tail distribution decays as fast as a Gaussian, namely,

$$\mathbb{P}(|z|\geq t)\leq 2\text{exp}\left\{-t^{2}/C^{2}\right\},\;\forall t\geq 0,$$

for some constant *C*.

A random vector $z\in {\mathbb{R}}^{m}$ is sub-Gaussian if **z**′**u** is sub-Gaussian for any $u\in {\mathbb{R}}^{m}$. The *ψ*_2_-norm of a vector **z** is defined as:

$${⦀z⦀}_{\psi_{2}}≜\underset{u\in S^{m}}{\text{sup}}{⦀z^{\prime }u⦀}_{\psi_{2}},$$

where $S^{m}$ denotes the unit sphere in the *m*-dimensional Euclidean space. For the properties of sub-Gaussian random variables/vectors, please refer to the book by Vershynin (2017).

#### Definition 2 (Gaussian width)

For any set $A\subseteq {\mathbb{R}}^{m}$, its Gaussian width is defined as:

(26)
$$w(A)≜E\left[\underset{u\in A}{\text{sup}}u^{\prime }g\right],$$

where g~N(0,I) is an m-dimensional standard Gaussian random vector.

### A.7 Proof of Theorem 3.6

In all the following proofs related to Section 3.2, we will adopt the notation **z** ≜ (**x**, *y*), **z**_*i*_ ≜ (**x**_*i*_, *y*_*i*_), $\tilde{\beta }≜(-\beta ,1)$, ${\tilde{\beta }}_{\text{est}}≜(-\hat{\beta },1)$, ${\tilde{\beta }}_{\text{true }}≜\left(-\beta^{*},1\right)$ for ease of exposition.

#### Proof

Since both $\hat{\beta }$ and ***β**** are feasible (the latter due to Assumption E), we have:

$${\left\|Z^{\prime }{\tilde{\beta }}_{\text{est}}\right\|}_{1}\leq \gamma_{N},$$

$${\left\|Z^{\prime }{\tilde{\beta }}_{\text{true}}\right\|}_{1}\leq \gamma_{N},$$

from which we derive that ${\left\|Z^{\prime }\left({\tilde{\beta }}_{\text{est }}-{\tilde{\beta }}_{\text{true }}\right)\right\|}_{1}\leq 2\gamma_{N}$. Since $\hat{\beta }$ is an optimal solution to (18) and ***β**** a feasible solution, it follows that ${\left\|{\tilde{\beta }}_{\text{est}}\right\|}_{*}\leq {\left\|{\tilde{\beta }}_{\text{true }}\right\|}_{*}$. This implies that $\nu ={\tilde{\beta }}_{\text{est}}-{\tilde{\beta }}_{\text{true}}$ satisfies the condition ${\left\|{\tilde{\beta }}_{\text{true}}+v\right\|}_{*}\leq {\left\|{\tilde{\beta }}_{\text{true}}\right\|}_{*}$ included in the definition of $A\left(\beta^{*}\right)$ and, furthermore, $\left({\tilde{\beta }}_{\text{est }}-{\tilde{\beta }}_{\text{true }}\right)/{\left\|{\tilde{\beta }}_{\text{est }}-{\tilde{\beta }}_{\text{true }}\right\|}_{2}\in A\left(\beta^{*}\right)$. Together with Assumption D, this yields

(27)
$${\left({\tilde{\beta }}_{\text{est }}-{\tilde{\beta }}_{\text{true }}\right)}^{\prime }ZZ^{\prime }\left({\tilde{\beta }}_{\text{est }}-{\tilde{\beta }}_{\text{true }}\right)\geq \underline{\alpha }{\left\|{\tilde{\beta }}_{\text{est }}-{\tilde{\beta }}_{\text{true }}\right\|}_{2}^{2}.$$

On the other hand, from the Cauchy-Schwarz inequality:

(28)
$${\left({\tilde{\beta }}_{\text{est }}-{\tilde{\beta }}_{\text{true }}\right)}^{\prime }ZZ^{\prime }\left({\tilde{\beta }}_{\text{est }}-{\tilde{\beta }}_{\text{true }}\right)\leq {\left\|Z^{\prime }\left({\tilde{\beta }}_{\text{est }}-{\tilde{\beta }}_{\text{true }}\right)\right\|}_{1}{\left\|Z^{\prime }\left({\tilde{\beta }}_{\text{est }}-{\tilde{\beta }}_{\text{true }}\right)\right\|}_{\infty }\;\leq 2\gamma_{N}\underset{i}{\text{max}}\left|z_{i}^{\prime }\left({\tilde{\beta }}_{\text{est }}-{\tilde{\beta }}_{\text{true }}\right)\right|\;\leq 2\gamma_{N}\underset{i}{\text{max}}{\left\|{\tilde{\beta }}_{\text{est }}-{\tilde{\beta }}_{\text{true }}\right\|}_{*}\left\|z_{i}\right\|\;\leq 2R\gamma_{N}{\left\|{\tilde{\beta }}_{\text{est }}-{\tilde{\beta }}_{\text{true }}\right\|}_{*}\;\text{.}$$

Combining (27) and (28), we have:

$${\left\|\hat{\beta }-\beta^{*}\right\|}_{2}={\left\|{\tilde{\beta }}_{\text{est }}-{\tilde{\beta }}_{\text{true }}\right\|}_{2}\;\leq \frac{2R\gamma_{N}}{\underline{\alpha }}\frac{{\left\|{\tilde{\beta }}_{\text{est }}-{\tilde{\beta }}_{\text{true }}\right\|}_{*}}{{\left\|{\tilde{\beta }}_{\text{est }}-{\tilde{\beta }}_{\text{true }}\right\|}_{2}}\;\leq \frac{2R\gamma_{N}}{\underline{\alpha }}\Psi \left(\beta^{*}\right),$$

where the last step follows from the fact that $\left({\tilde{\beta }}_{\text{est }}-{\tilde{\beta }}_{\text{true }}\right)/{\left\|{\tilde{\beta }}_{\text{est }}-{\tilde{\beta }}_{\text{true }}\right\|}_{2}\in A\left(\beta^{*}\right)$. ■

### A.8 Proof of Lemma 3.7

#### Proof

Define $\hat{\Gamma }=\frac{1}{N}\sum_{i=1}^{N}Z_{i}z_{i}^{\prime }$. Consider the set of functions $F=\{f_{w}(z)=z^{\prime }\Gamma^{-1/2}w|w\in A_{\Gamma }\}$. Then, for any $f_{w}\in F$,

$$E\left[f_{\text{w}}^{2}\right]=E\left[w^{\prime }\Gamma^{-1/2}{\text{zz}}^{\prime }\Gamma^{-1/2}w\right]\;=w^{\prime }\Gamma^{-1/2}E\left[zz^{\prime }\right]\Gamma^{-1/2}w\;=w^{\prime }w\;\text{=1,}$$

where we used $\Gamma =E\left[zz^{\prime }\right]$ and the fact that $w\in A_{\Gamma }$.

For any $f_{w}\in F$ we have

$${⦀f_{w}⦀}_{\psi_{2}}=⦀{z^{\prime }\Gamma^{-1/2}w⦀}_{\psi_{2}}\;={⦀z^{\prime }\Gamma^{-1/2}w⦀}_{\psi_{2}}\frac{{\left\|\Gamma^{-1/2}w\right\|}_{2}}{{\left\|\Gamma^{-1/2}w\right\|}_{2}}\;={⦀z^{\prime }\frac{\Gamma^{-1/2}w}{{\left\|\Gamma^{-1/2}w\right\|}_{2}}⦀}_{\psi_{2}}{\left\|\Gamma^{-1/2}w\right\|}_{2}\;\leq \mu \sqrt{w^{\prime }\Gamma^{-1}w}\;\leq \mu \sqrt{\frac{1}{\lambda_{\text{min}}}\|w{\|}_{2}^{2}}\;=\mu \sqrt{\frac{1}{\lambda_{\text{min}}}}=\bar{\mu },$$

where the first inequality used Assumption F and the second inequality used Assumption G.

Applying Theorem D from Mendelson et al. (2007), for any *θ >* 0 and when

$${\tilde{C}}_{1}\bar{\mu }\gamma_{2}\left(F,{⦀\cdot ⦀}_{\psi_{2}}\right)\leq \theta \sqrt{N},$$

with probability at least $1-\text{exp}\left(-{\tilde{C}}_{2}\theta^{2}N/{\bar{\mu }}^{4}\right)$ we have

(29)
$$\underset{f_{\text{w}}\in F}{\text{sup}}\left|\frac{1}{N}\sum_{i=1}^{N}f_{w}^{2}\left(z_{i}\right)-E\left[f_{w}^{2}\right]\right|=\underset{f_{w}\in F}{\text{sup}}\left|\frac{1}{N}\sum_{i=1}^{N}w^{\prime }\Gamma^{-1/2}z_{i}z_{i}^{\prime }\Gamma^{-1/2}w-1\right|\;=\underset{w\in A_{\Gamma }}{\text{sup}}\left|w^{\prime }\Gamma^{-1/2}\hat{\Gamma }\Gamma^{-1/2}w-1\right|\;\leq \theta ,$$

where ${\tilde{C}}_{1}$ is some positive constant and $\gamma_{2}\left(F,{⦀\cdot ⦀}_{\psi_{2}}\right)$ is defined in Mendelson et al. (2007) as a measure of the size of the set F with respect to the metric ${⦀\cdot ⦀}_{\psi_{2}}$. Using *θ* = 1/2, and properties of $\gamma_{2}\left(F,{⦀\cdot ⦀}_{\psi_{2}}\right)$ outlined in Chen and Banerjee (2016), we can set *N* to satisfy

$${\tilde{C}}_{1}\bar{\mu }\gamma_{2}\left(F,⦀\cdot {⦀}_{\psi_{2}}\right)\leq {\tilde{C}}_{1}{\bar{\mu }}^{2}\gamma_{2}\left(A_{\Gamma },\|\cdot {\|}_{2}\right)\;\leq {\tilde{C}}_{1}{\bar{\mu }}^{2}C_{0}w\left(A_{\Gamma }\right)\;\leq \frac{1}{2}\sqrt{N},$$

for some positive constant *C*_0_, where we used Eq. (44) in Chen and Banerjee (2016). This implies

$$N\geq C_{1}{\bar{\mu }}^{4}{\left(w\left(A_{\Gamma }\right)\right)}^{2}$$

for some positive constant *C*_1_. Thus, for such *N* and with probability at least $1-\text{exp}\left(-C_{2}N/{\bar{\mu }}^{4}\right)$, for some positive constant *C*_2_, (29) holds with *θ* = 1/2. This implies that for all $w\in A_{\Gamma }$,

$$\left|w^{\prime }\Gamma^{-1/2}\hat{\Gamma }\Gamma^{-1/2}w-1\right|\leq \frac{1}{2}$$

or

$$w^{\prime }\Gamma^{-1/2}\hat{\Gamma }\Gamma^{-1/2}w\geq \frac{1}{2}=\frac{1}{2}w^{\prime }\Gamma^{-1/2}\Gamma \Gamma^{-1/2}w.$$

By the definition of $A_{\Gamma }$, for any $v\in A\left(\beta^{*}\right)$,

$$v^{\prime }\hat{\Gamma }v\geq \frac{1}{2}v^{\prime }\Gamma v.$$

Noting that $\hat{\Gamma }=(1/N)ZZ^{\prime }$ yields the desired result. ■

### A.9 Proof of Lemma 3.8

We follow the proof of Lemma 4 in Chen and Banerjee (2016), adapted to our setting. We include all key steps for completeness.

#### Proof

Recall the definition of the Gaussian width $w\left(A_{\Gamma }\right)$ (cf. (26)):

$$w\left(A_{\Gamma }\right)=E\left[\underset{u\in A_{\Gamma }}{\text{sup}}u^{\prime }g\right],$$

where g~N(0,I). We have:

$$\underset{w\in A_{\Gamma }}{\text{sup}}w^{\prime }g=\underset{w\in A_{\Gamma }}{\text{sup}}w^{\prime }\Gamma^{-1/2}\Gamma^{1/2}g\;=\underset{w\in A_{\Gamma }}{\text{sup}}{\left\|\Gamma^{-1/2}w\right\|}_{2}\frac{w^{\prime }\Gamma^{-1/2}}{{\left\|\Gamma^{-1/2}w\right\|}_{2}}\Gamma^{1/2}g\;\leq \sqrt{\frac{1}{\lambda_{\text{min}}}}\underset{v\in \text{cone}\left(A\left(\beta^{*}\right)\right)\cap B^{m}}{\text{sup}}v^{\prime }\Gamma^{1/2}g,$$

where $B^{m}$ is the unit ball in the *m*-dimensional Euclidean space and the inequality used Assumption G and the fact that $w^{\prime }\Gamma^{-1/2}/{\left\|\Gamma^{-1/2}w\right\|}_{2}\in B^{m}$ and $w\in A_{\Gamma }$.

Define $T=\text{cone}\left(A\left(\beta^{*}\right)\right)\cap B^{m}$, and consider the stochastic process ${\left\{S_{v}=v^{\prime }\Gamma^{1/2}g\right\}}_{v\in T}$. For any **v**_1_, $v_{2}\in T$,

$${⦀S_{v_{1}}-S_{v_{2}}⦀}_{\psi_{2}}={⦀{\left(v_{1}-v_{2}\right)}^{\prime }\Gamma^{1/2}g⦀}_{\psi_{2}}\;={\left\|\Gamma^{1/2}\left(v_{1}-v_{2}\right)\right\|}_{2}{⦀\frac{{\left(v_{1}-v_{2}\right)}^{\prime }\Gamma^{1/2}g}{{\left\|\Gamma^{1/2}\left(v_{1}-v_{2}\right)\right\|}_{2}}⦀}_{\psi_{2}}\;\leq {\left\|\Gamma^{1/2}\left(v_{1}-v_{2}\right)\right\|}_{2}\underset{u\in S^{m}}{\text{sup}}{⦀u^{\prime }g⦀}_{\psi_{2}}\;=\mu_{0}{\left\|\Gamma^{1/2}\left(v_{1}-v_{2}\right)\right\|}_{2}\;\leq \mu_{0}\sqrt{\lambda_{\text{max}}}{\left\|v_{1}-v_{2}\right\|}_{2},$$

where the last step used Assumption G.

Then, by the tail behavior of sub-Gaussian random variables (see Hoeffding bound, Thm. 2.6.2 in (Vershynin, 2017)), we have:

$$\mathbb{P}\left(\left|S_{v_{1}}-S_{v_{2}}\right|\geq \delta \right)\leq 2\text{exp}\left(-\frac{C_{01}\delta^{2}}{\mu_{0}^{2}\lambda_{\text{max}}{\left\|v_{1}-v_{2}\right\|}_{2}^{2}}\right),$$

for some positive constant *C*_01_.

To bound the supremum of *S*_**v**_, we define the metric $s\left(v_{1},v_{2}\right)=\mu_{0}\sqrt{\lambda_{\text{max}}}{\left\|v_{1}-v_{2}\right\|}_{2}$. Then, by Lemma B in Chen and Banerjee (2016),

$$E\left[\underset{v\in T}{\text{sup}}v^{\prime }\Gamma^{1/2}g\right]\leq C_{02}\gamma_{2}(T,s)\;=C_{02}\mu_{0}\sqrt{\lambda_{\text{max}}}\gamma_{2}\left(T,\|\cdot {\|}_{2}\right)\;\leq C_{3}\mu_{0}\sqrt{\lambda_{\text{max}}}w(T),$$

for positive constants *C*_02_, *C*_3_, where $\gamma_{2}(T,s)$ is the *γ*_2_-functional we referred to in the proof of Lemma 3.7. Since $T=\text{cone}\left(A\left(\beta^{*}\right)\right)\cap B^{m}\subseteq \text{conv}\left(A\left(\beta^{*}\right)\cup \{0\}\right)$, by Lemma 2 in Maurer et al. (2014),

$$w(T)\leq w\left(\text{conv}\left(A\left(\beta^{*}\right)\cup \{0\}\right)\right)\;=w\left(A\left(\beta^{*}\right)\cup \{0\}\right)\;\leq \text{max}\left\{w\left(A\left(\beta^{*}\right)\right),w(\{0\})\right\}+2\sqrt{\text{ln}4}\;\leq w\left(A\left(\beta^{*}\right)\right)+3.$$

Thus,

$$w\left(A_{\Gamma }\right)=E\left[\underset{w\in A_{\Gamma }}{\text{sup}}w^{\prime }g\right]\;\leq \sqrt{\frac{1}{\lambda_{\text{min}}}}E\left[\underset{v\in T}{\text{sup}}v^{\prime }\Gamma^{1/2}g\right]\;\leq C_{3}\sqrt{\frac{1}{\lambda_{\text{min}}}}\mu_{0}\sqrt{\lambda_{\text{max}}}w(T)\;\leq C_{3}\mu_{0}\sqrt{\frac{\lambda_{\text{max}}}{\lambda_{\text{min}}}}\left(w\left(A\left(\beta^{*}\right)\right)+3\right).$$

### A.10 Proof of Corollary 3.9

#### Proof

Combining Lemmas 3.7 and 3.8, and using the fact that for any $v\in A\left(\beta^{*}\right)$,

$$\frac{N}{2}v^{\prime }\Gamma v\geq \frac{N\lambda_{\text{min}}}{2},$$

we can derive the desired result. ■

### A.11 Proof of Lemma 3.10

#### Proof

By the definition of dual norm, we know that:

$${\left\|{\tilde{\beta }}^{\prime }Z\right\|}_{1}=\underset{v\in B_{u}}{\text{sup}}{\tilde{\beta }}^{\prime }Zv=\underset{v\in B_{u}}{\text{sup}}\sum_{i=1}^{N}v_{i}{\tilde{\beta }}^{\prime }z_{i}.$$

Since $v_{i}{\tilde{\beta }}^{\prime }z_{i}$, *i* = 1, … , *N* are independent centered sub-Gaussian random variables, and

$${⦀v_{i}{\tilde{\beta }}^{\prime }z_{i}⦀}_{\psi_{2}}\leq \mu {\left\|v_{i}\tilde{\beta }\right\|}_{2},$$

we have that $\sum_{i=1}^{N}v_{i}{\tilde{\beta }}^{\prime }z_{i}$ is also a centered sub-Gaussian random variable with

$${⦀\sum_{i=1}^{N}v_{i}{\tilde{\beta }}^{\prime }z_{i}⦀}_{\psi_{2}}^{2}\leq C_{03}^{2}\sum_{i=1}^{N}\mu^{2}{\left\|v_{i}\tilde{\beta }\right\|}_{2}^{2}\;=C_{03}^{2}\mu^{2}\|\tilde{\beta }{\|}_{2}^{2}\|v{\|}_{2}^{2},$$

for a positive constant *C*_03_.

Consider the stochastic process ${\left\{S_{v}={\tilde{\beta }}^{\prime }Zv\right\}}_{v\in B_{u}}$. As in the proof of Lemma 3.8,

$${⦀S_{v_{1}}-S_{v_{2}}⦀}_{\psi_{2}}\leq C_{03}\mu \|\tilde{\beta }{\|}_{2}{\left\|v_{1}-v_{2}\right\|}_{2}.$$

By the tail behavior of sub-Gaussian random variables (Vershynin, 2017), we know:

$$\mathbb{P}\left(\left|S_{v_{1}}-S_{v_{2}}\right|\geq \delta \right)\leq 2\;\text{exp}\left(-\frac{C_{04}\delta^{2}}{\mu^{2}\|\tilde{\beta }{\|}_{2}^{2}{\left\|v_{1}-v_{2}\right\|}_{2}^{2}}\right),$$

for a positive constant *C*_04_.

Define the metric $s\left(v_{1},v_{2}\right)=\mu \|\tilde{\beta }{\|}_{2}{\left\|v_{1}-v_{2}\right\|}_{2}$. Then, by Lemma B in Chen and Banerjee (2016),

$$\mathbb{P}\left(\underset{v_{1},v_{2}\in B_{u}}{\text{sup}}\left|S_{v_{1}}-S_{v_{2}}\right|\geq C_{05}\left(\gamma_{2}\left(B_{u},s\right)+\delta \cdot \text{diam}\left(B_{u},s\right)\right)\right)\leq C_{4}\text{exp}\left(-\delta^{2}\right),$$

for positive constants *C*_05_, *C*_4_. Also,

$$\gamma_{2}\left(B_{u},s\right)=\mu \|\tilde{\beta }{\|}_{2}\gamma_{2}\left(B_{u},\|\cdot {\|}_{2}\right)\leq C_{5}\mu \|\tilde{\beta }{\|}_{2}w\left(B_{u}\right),\;\text{diam}\left(B_{u},s\right)=\underset{v_{1},v_{2}\in B_{u}}{\text{sup}}s\left(v_{1},v_{2}\right)\;=\mu \|\tilde{\beta }{\|}_{2}\underset{v_{1},v_{2}\in B_{u}}{\text{sup}}{\left\|v_{1}-v_{2}\right\|}_{2}\;\leq 2\mu \|\tilde{\beta }{\|}_{2}\underset{v\in B_{u}}{\text{sup}}\|v{\|}_{2}\;=2\mu \|\tilde{\beta }{\|}_{2}\rho ,$$

for positive constants *C*_5_. Therefore, noting that ${\text{sup}}_{v_{1},v_{2}\in B_{u}}\left|S_{v_{1}}-S_{v_{2}}\right|\geq 2\;{\text{sup}}_{v\in B_{u}}S_{v}$, we obtain

$$\mathbb{P}\left(\underset{v\in B_{u}}{\text{sup}}S_{v}\geq C_{05}\left(\frac{C_{5}}{2}\mu \|\tilde{\beta }{\|}_{2}w\left(B_{u}\right)+\delta \mu \|\tilde{\beta }{\|}_{2}\rho \right)\right)\;\leq \mathbb{P}\left(\underset{v_{1},v_{2}\in B_{u}}{\text{sup}}\left|S_{v_{1}}-S_{v_{2}}\right|\geq C_{05}\left(\gamma_{2}\left(B_{u},s\right)+\delta \text{diam}\left(B_{u},s\right)\right)\right)\;\leq C_{4}\text{exp}\left(-\delta^{2}\right).$$

Set $\delta =\frac{C_{5}w\left(B_{u}\right)}{2\rho }$; then with probability at least $1-C_{4}\text{exp}\left(-\frac{C_{5}^{2}{\left(w\left(B_{u}\right)\right)}^{2}}{4\rho^{2}}\right)$,

$$\underset{v\in B_{u}}{\text{sup}}S_{v}\leq C\mu {\bar{B}}_{2}w\left(B_{u}\right).$$

The result follows. ■

### A.12 Proof of the Result in Section 4

We will show that if the Wasserstein metric is defined by the following metric *s*_*c*_:

$$s_{c}(x,y)=\|(x,cy){\|}_{\infty },$$

then as *c* → ∞, the corresponding Wasserstein DRO formulation becomes:

$$\underset{\beta \in B}{\text{inf}}\frac{1}{N}\sum_{i=1}^{N}\left|y_{i}-x_{i}^{\prime }\beta \right|+\epsilon \|\beta {\|}_{1},$$

which is the *ℓ*_1_-regularized LAD.

#### Proof

We first define a new notion of norm on (**x**, *y*) where **x** = (*x*_1_, … , *x*_*m*−1_):

$$\|(x,y){\|}_{w,p}≜{\left\|\left(x_{1}w_{1},\ldots ,x_{m-1}w_{m-1},yw_{m}\right)\right\|}_{p},$$

for some *m*-dimensional weighting vector **w** = (*w*_1_, … , *w*_*m*_), and *p* ≥ 1. Then, *s*_*c*_(**x**, *y*) = ‖(**x**, *y*)‖_**w**,∞_ with **w** = (1, … , 1, *c*). To obtain the Wasserstein DRO formulation, the key is to derive the dual norm of ‖ · ‖_**w**,∞_. Hölder’s inequality (Rogers, 1888) will be used for the derivation. We state it below for convenience.

#### Theorem 1 (Hölder’s inequality)

Suppose we have two scalars *p*, *q >* 1 *and* 1/*p*+1/*q* = 1. For any two vectors **a** = (*a*_1_, … , *a*_*n*_) and **b** = (*b*_1_, … , *b*_*n*_), the following holds.

$$\sum_{i=1}^{n}\left|a_{i}b_{i}\right|\leq \|a{\|}_{p}\|b{\|}_{q}.$$

We will use the notation **z** ≜ (**x**, *y*). Based on the definition of dual norm, we are interested in solving the following optimization problem for $\tilde{\beta }\in {\mathbb{R}}^{m}$:

(30)
$$\begin{array}{l} \underset{z}{\text{max}}\;z^{\prime }\tilde{\beta }\\ \text{s.t.}\;\|Z{\|}_{w,\infty }\leq 1\;\text{.} \end{array}$$

The optimal value of problem (30), which is a function of $\tilde{\beta }$, gives the dual norm evaluated at $\tilde{\beta }$. Using Hölder’s inequality, we can write

$$z^{\prime }\tilde{\beta }=\sum_{i=1}^{m}\left(w_{i}z_{i}\right)\left(\frac{1}{w_{i}}{\tilde{\beta }}_{i}\right)\leq \|z{\|}_{w,\infty }\|\tilde{\beta }{\|}_{w^{-1},1}\leq \|\tilde{\beta }{\|}_{w^{-1},1},$$

where $w^{-1}≜\left(\frac{1}{w_{1}},\ldots ,\frac{1}{w_{m}}\right)$. The last inequality is due to the constraint ‖**z**‖_**w**,∞_ ≤ 1. It follows that the dual norm of ‖ · ‖_**w**,∞_ is just $\|\cdot {\|}_{{\text{w}}^{-1},1}$. Back to our problem setting, using **w** = (1, … , 1, *c*), and evaluating the dual norm at (−***β***, 1), we have the following Wasserstein DRO formulation as *c* → ∞:

$$\underset{c\to \infty }{\text{lim}}\underset{\beta \in B}{\text{inf}}\frac{1}{N}\sum_{i=1}^{N}\left|y_{i}-{\text{x}}_{i}^{\prime }\beta \right|+\epsilon \|(-\beta ,1){\|}_{w^{-1},1}=\underset{\beta \in B}{\text{inf}}\frac{1}{N}\sum_{i=1}^{N}\left|y_{i}-x_{i}^{\prime }\beta \right|+\epsilon \|\beta {\|}_{1}.$$

■
